# DLoopCaller: A deep learning approach for predicting genome-wide chromatin loops by integrating accessible chromatin landscapes

**DOI:** 10.1371/journal.pcbi.1010572

**Published:** 2022-10-07

**Authors:** Siguo Wang, Qinhu Zhang, Ying He, Zhen Cui, Zhenghao Guo, Kyungsook Han, De-Shuang Huang

**Affiliations:** 1 Institute of Machine Learning and Systems Biology, School of Electronics and Information Engineering, Tongji University, Shanghai, China; 2 Translational Medical Center for Stem Cell Therapy and Institute for Regenerative Medicine, Shanghai East Hospital, Bioinformatics Department, School of Life Sciences and Technology, Tongji University, Shanghai, China; 3 The School of Computer Science and Engineering, Inha University, Incheon, South Korea; 4 EIT Institute for Advanced Study, Zhenhai, Ningbo, Zhejiang, China; 5 Big Data and Intelligent Computing Research Center, Guangxi Academy of Science, Nanning, China; La Jolla Institute for Allergy and Immunology, UNITED STATES

## Abstract

In recent years, major advances have been made in various chromosome conformation capture technologies to further satisfy the needs of researchers for high-quality, high-resolution contact interactions. Discriminating the loops from genome-wide contact interactions is crucial for dissecting three-dimensional(3D) genome structure and function. Here, we present a deep learning method to predict genome-wide chromatin loops, called DLoopCaller, by combining accessible chromatin landscapes and raw Hi-C contact maps. Some available orthogonal data ChIA-PET/HiChIP and Capture Hi-C were used to generate positive samples with a wider contact matrix which provides the possibility to find more potential genome-wide chromatin loops. The experimental results demonstrate that DLoopCaller effectively improves the accuracy of predicting genome-wide chromatin loops compared to the state-of-the-art method Peakachu. Moreover, compared to two of most popular loop callers, such as HiCCUPS and Fit-Hi-C, DLoopCaller identifies some unique interactions. We conclude that a combination of chromatin landscapes on the one-dimensional genome contributes to understanding the 3D genome organization, and the identified chromatin loops reveal cell-type specificity and transcription factor motif co-enrichment across different cell lines and species.

## Author summary

The emergence of chromosome conformation capture technologies has provided researchers with the opportunity to understand the role of three-dimensional genome structure in regulating gene expression and cell functions. Although significant progress has been made in studying the basic functional units (called chromatin loops) that directly regulate gene expression, but still have limitations on how to adequately extract features from the contact maps and rationally utilize multi-omics data. In this work, we effectively combine accessible chromatin landscapes and raw Hi-C contact maps data based on a deep learning framework to identify genome-wide chromatin loops. Besides, we use some available orthogonal data ChIA-PET/HiChIP and Capture Hi-C were used to generate training samples. We demonstrate the performance of our proposed method to identify some unique chromatin loops with high confidence. Moreover, the identified chromatin loops further reveal cell-type specificity and transcription factor motif co-enrichment across different cell lines and species, which may help us understand the mechanism of tissue-specific gene expression and transcriptional regulation.

This is a *PLOS Computational Biology* Methods paper.

## Introduction

In eukaryotes, chromatin is folded into complex 3D structures and dynamically regulates the life processes. Therefore, dissecting the rules that govern chromatin dynamics is essential to comprehend the tissue-specific gene regulation, which provides the rationale for understanding the role of noncoding region variants associated with disease [1–3]. In the past two decades, many high-throughput technologies have emerged for researchers to reveal the significance of chromatin structure for gene regulatory networks. According to these technologies, from a genome-wide perspective, the multiscale high-dimensional chromatin structure is divided into A/B compartments, more refined nuclear compartmentalization, topologically associating domains (TADs), and chromatin loops [4–9]. Gene regulatory networks rely on cis-regulatory elements, for example, many enhancers function over long genomic distances to regulate gene expression by forming topological loops with distant promoters and form an active chromatin hub consisting of multiple enhancers and their interacting promoters [10–12]. In addition, ChIA-PET data indicate that architectural proteins play an important role in forming chromatin structure and regulating transcription, including CCCTC-binding factor (CTCF), cohesin, and RNA polymerase II [13]. Sanborn et al. revealed that chromatin loops are mediated by two pairs of structural proteins CTCF and cohesin in a loop extrusion model, and until the corresponding CTCF is detected on the strand will stop [14]. The spatial chromatin structure is not only characterized by gene expression but also conserved across species [15]. Although several studies have given significant insights into 3D genome organization and function, they still lack of capacity to describe chromatin loops in the 3D space of the nucleus and predict the impact of structural changes on genetic mutations. Understanding the relationship between the complex structure and function of the genome remains a big challenge, hence more computational models are urgently needed to be proposed for 3D genomic studies.

Numerous experimental methods have been developed to predict 3D chromatin loops, mainly divided into the following aspects: (1) Sequencing-based techniques. High-throughput chromosome conformation capture (Hi-C) aims to sequence 3D interactions at the genome-wide level, which include dilution during proximity ligation but is less effective [7,8]. The ensuing in situ ligation compensates for the deficiencies, efficiently capturing true contacts and providing higher resolution at the same sequencing depth. GAM and SPRITE are used to analyze two-way and multi-way contacts, enabling the direct study of multivalent enhancer–promoter interactions [16,17]. However, to finely map chromatin folding and understand some of its functional aspects, it is necessary to detect specific contacts using enrichment methods that amplify the contact signal in specific genomic regions of interest. Other 3C-based technologies have been proposed, Capture-HiC technology captures chromatin interaction maps in specific regions (such as promoter regions) through hybridization probes, which is low-cost but achieves deeper-depth sequencing [18]. And some interaction maps are mediated by the specific proteins of interest, such as Chromatin Interaction Analysis with Paired-End Tag sequencing (ChIA-PET) [19] and HiChIP [20]. (2) Super-resolution microscopy methods. Stochastic optical reconstruction microscopy (STORM) [21] and Structured illumination microscopy (SIM) [22] are two classical methods to illustrate the power of single-cell super-resolution imaging.

With the advent of Hi-C and related technologies, some computational analysis tools, Fit-Hi-C [23] and HiCCUPS [8,24] are the two most popular enrichment-based methods, have been proposed to study the inherent complexity of Hi-C data. Fit-Hi-C model the random polymer looping effect to assign statistical confidence with genomic distance into account, specific chromatin contacts remarkably increase about contact detection compared with general background model. HiCCUPS identifies “enriched pixels” as chromatin loops which means comparing the number of contacts in the pixel with a series of regions surrounding the pixel. Although these computational tools have made great progress, they still have some drawbacks, such as high cost and conservative. All of these limitations have stimulated the development of computational analyses and mathematical models, combined with experimental methods, which may quantitatively and predictively understand chromosome structure and function. For example, CHiCAGO applies a convolution background model to predict DNA looping interactions in Capture Hi-C data [25]. To date, there are some studies to predict CTCF-mediated chromatin interactions based on a random forest model by integrating genomic, epigenomic features, or transcription factor profiles [26,27]. Owing to the rapid development and widespread application of deep learning techniques, it is not surprising that significant progress has been made in bioinformatics [28–32], and some works have been made in the field of genomics. For instance, Deep-loop predicted CTCF-mediated chromatin loops and performs well in different cell lines [33], and DeepMILO predicted the effects of variants on CTCF and cohesin-mediated insulator loops based on a deep learning framework [34]. Furthermore, Mustache employed scale-space theory in computer vision to detect chromatin loops in contact maps, regarding only locally enriched pixels as loops [35]. It is worth noting that Peakachu built a random forest to predict chromatin loops in genome-wide contact maps, which transforms the task of detecting chromatin loops into a binary classification problem by using biologically enriched experiments such as ChIA-PET/HiChIP and Capture Hi-C as positive samples and non-interaction regions as negative samples, achieving impressive prediction performance [36].

**Fig 1:**
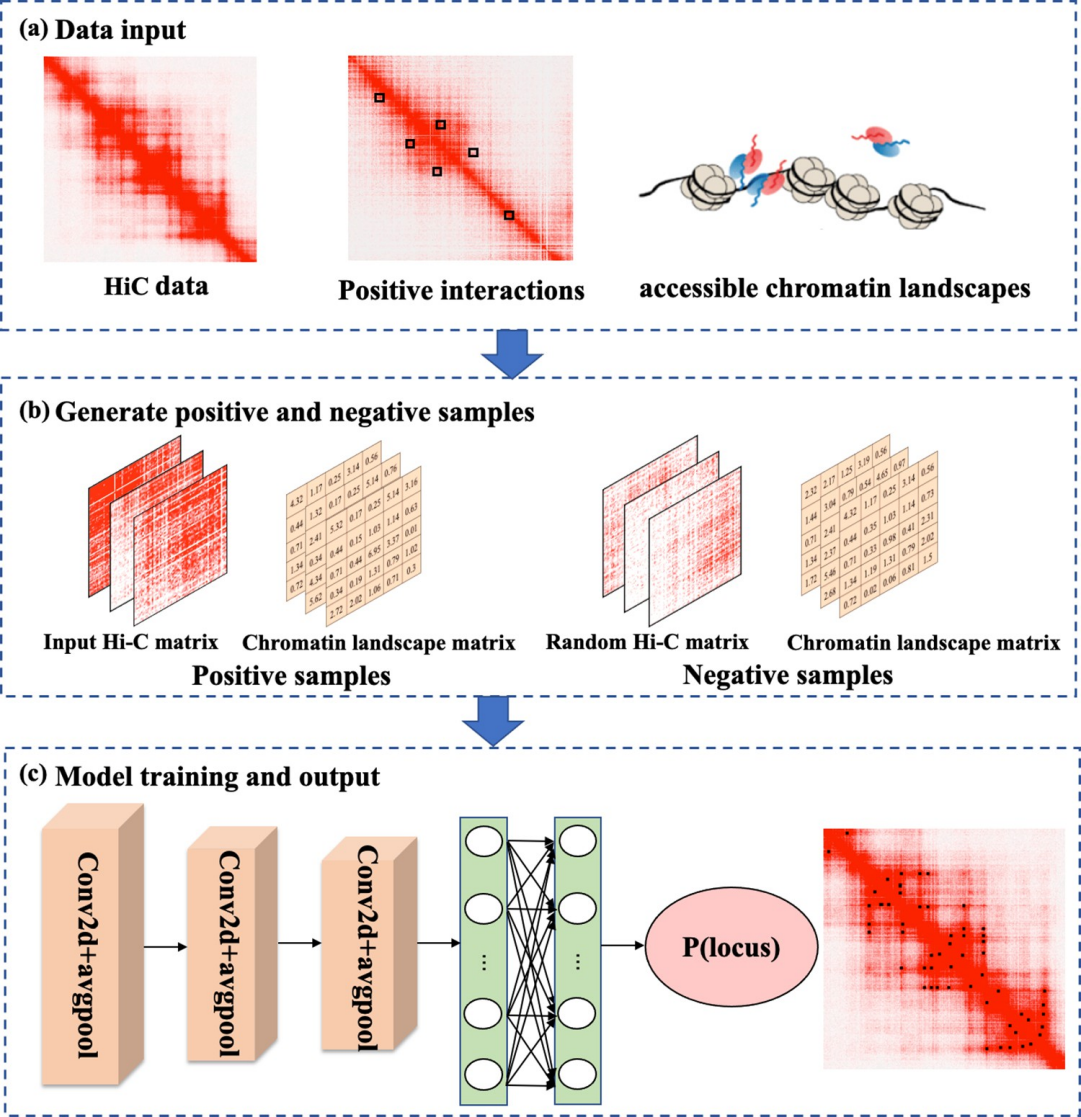
The workflow of DLoopCaller. (a) Data inputs includes Hi-C matrix, accessible chromatin landscapes, and enriched experimental data such as ChIA-PET/HiChIP and Capture Hi-C as positive interactions. (b) Positive samples are generated according to the input data, and negative samples are generated according to the similar distance or greater distance of the positive samples. (c) DLoopCaller includes three convolutional blocks, two fully connected layers and a classification layer, in which each block consists of a convolutional layer, a ReLU layer, a dropout layer, and followed by a global average pooling layer.

Although these methods have achieved breakthroughs, how to adequately extract features from the contact maps and rationally utilize multi-omics data to identify chromatin loops is still a big challenge. In this study, we present a new method named DLoopCaller, based on a deep learning framework, for predicting chromatin loops in genome-wide contact maps by integrating raw Hi-C matrix and accessible chromatin landscape. Similar to Peakachu, DLoopCaller transforms the task of detecting chromatin loops into a binary classification problem by using enriched experimental data such as ChIA-PET/HiChIP and Capture Hi-C as positive interactions and non-interaction regions as negative samples. The contributions of DLoopCaller mainly include the following aspects: (i) efficiently combining one dimensional (1D) open chromatin landscapes with 3D genomic data for chromatin loops prediction; (ii) improving the identification accuracy of chromatin loops on wider chromatin contact matrix; (iii) and compared with some existing methods, our method identifies a series of unique chromatin loops at 10 kb in genome-wide contact maps; (iv) the identified chromatin loops reveal cell-type specificity and transcription factor motif co-enrichment; (v) DLoopCaller is robust and reproducible to some extent. The workflow of DLoopCaller is shown in Fig 1.

## Materials and methods

### Data collection and preprocessing

We performed experiments on four cell lines, including K562 (chronic myelogenous leukemia), GM12878 (lymphoblastoid cell), H1-ESC (hematopoietic stem cell), and mESC (mouse embryonic stem cells), and the data inputs include Hi-C data, accessible chromatin data, and corresponding enriched experimental data. The original Hi-C data were converted into 10kb resolution contact matrices and normalized by using hic2cool and cooler python package.

The Hi-C contact maps of GM12878 can be downloaded from https://drive.google.com/file/d/1rfkdHSfmn5GK7qdzSwVlrSHpJVPPn5R3/view?usp=sharing. In order to reduce data bias, we merged the accessible chromatin landscapes of two replicate samples as the final data of GM12878, which were obtained from ENCODE with accession code ENCFF264NMW and ENCFF901GZH. The enriched experimental data in GM12878 include CTCF ChIA-PET interactions [13], Rad21 ChIA-PET interactions [37], SMC1 HiChIP interactions [20], H3K27ac HiChIP interactions [1] and promoter Capture Hi-C interactions [25].

The Hi-C contact maps of K562 were obtained from the ENCODE with accession code ENCFF013TGD (replicate1) and ENCFF097SKJ (replicate2). The accessible chromatin landscape of K562 was obtained from ENCODE with accession code ENCFF352SET. The enriched experimental data CTCF ChIA-PET interactions in K562 were obtained from ENCODE with accession code ENCFF001THV.

The Hi-C contact maps of H1-ESC were obtained from the 4DN data portal with accession code 4DNFI6HDY7W. The accessible chromatin landscape of H1-ESC was obtained from the 4DN data portal with accession code 4DNFIQNCHGRE. The enriched experimental data CTCF ChIA-PET interactions in H1-ESC were obtained from the 4DN data portal with accession code 4DNESR9S8R38.

The Hi-C contact maps of mESC were obtained from ENCODE with accession code ENCFF289WNN. The accessible chromatin landscape of the mESC was obtained from NCBI with accession code GSE137335. The enriched experimental data SMC1 HiChIP interactions in mESC were obtained from [20].

All mentioned positive interactions obtained from enrichment experiments are consistent with Peakachu, provided at https://github.com/wangguoguoa/DLoopCaller/tree/main/training-sets. The enhancer and promoter loci in GM12878, K562, H1-ESC provided at https://github.com/wangguoguoa/DLoopCaller/tree/main/annotations, which were extracted from public ChromHMM annotations in ENCODE.

## Methods

### The generation of training samples

**Fig 2:**
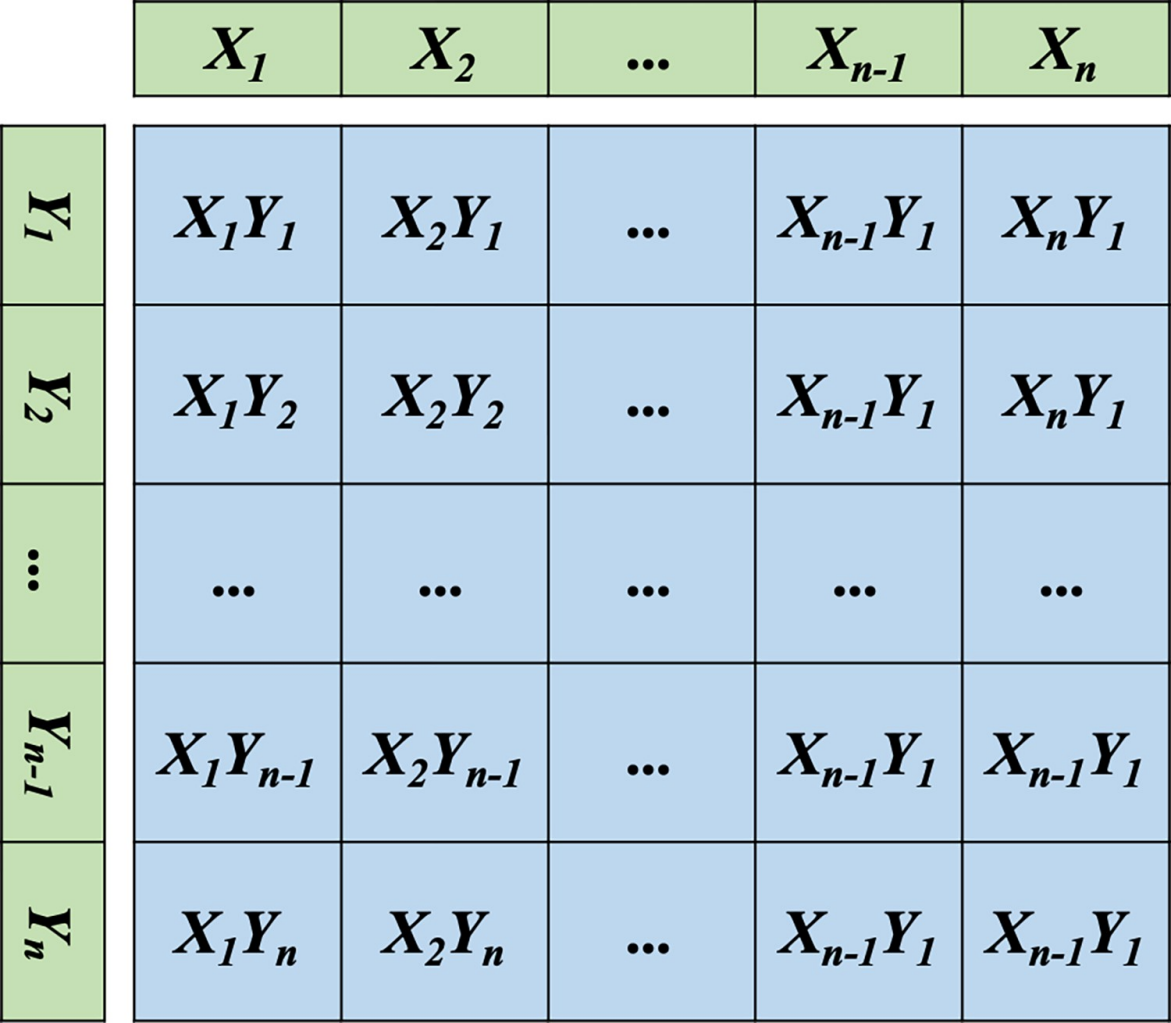
The generation of accessible chromatin matrix.

The data inputs of DLoopCaller mainly include three parts: the original Hi-C matrix, some verified positive interactions involving targeted regions or proteins of interest by biologically enriched experiments such as ChIA-PET/HiChIP and Capture Hi-C, and the corresponding accessible chromatin landscapes, which were then used to generate training samples for training model. Briefly, (i) The pixels around each positive interaction were used as the features of the training samples, in which the pixel of the positive interaction was expanded along both sides in the raw HiC matrix to obtain a 23*23 positive Hi-C matrix; (ii) In order to obtain the corresponding accessible chromatin matrix, the 1D accessible chromatin data were firstly averaged at every 10kb distance to keep the resolution consistency and reduce the data deviation. Then the chromatin accessible data of the *x*-axis peak loci and *y*-axis peak loci in the positive HiC matrix were used to obtain the positive accessible chromatin matrix by Cartesian product. For example, *x* = {*X*_*1*_, *X*_*2*_*… X*_*n*_} and *y* = {*X*_*1*_, *X*_*2*_*… X*_*n*_}, the accessible chromatin matrix is defined as follows (Fig 2):

where *n* = 23 and the blue matrix is the accessible chromatin matrix; (iii) the negative Hi-C matrix with an equal number of pixels from nonzero values was randomly sampled from two aspects: (1) matching the similar distance of positive interactions according to the probability density function of the distance; (2) considering greater distance, larger than maximum distance of the positive interactions, to improve the diversity of negative samples. Similarly, we obtained the corresponding accessible chromatin matrix for the negative HiC matrix following the same way described above. And we list the number of samples in each dataset in S1 Table.

### The framework of neural network architecture

Some studies have shown that three-layer convolutional neural networks (CNN) are sufficient to mine features from complex biological data to achieve good experimental results [38–41]. Therefore, DLoopCaller applied a three-layer CNN model to extract features from the generated Hi-C matrix and accessible chromatin landscape matrix and retained the best training model for identifying genome-wide chromatin loops. As shown in Fig 1, DLoopCaller takes two-channel as input into the model, inspired by the way of image processing research. To reduce data bias and noise, the positive/ negative Hi-C matrix and accessible chromatin landscape matrix were normalized before training. The normalization of each matrix is as follows:

M(x,y)←log10(1+M(x,y))

$$M(x,y)\leftarrow \frac{M(x,y)}{1+max(M(x,y))}$$ (1)

where *x*, *y* refers to the coordinates of the Hi-C matrix or the accessible chromatin landscape matrix *M*, respectively, max (*M*(*x,y*)) denotes the maximum value in the corresponding positive/negative Hi-C matrix and accessible chromatin landscape matrix.

The framework of DLoopCaller is composed of three convolutional blocks, in which each block consists of a convolutional layer, a ReLU layer, a dropout layer, and a global average pooling layer. The convolutional layer is used to directly capture local features and the global average pooling layer is used to capture the global textual information from the Hi-C matrix and chromatin landscape matrix, and the dropout layer is used to avoid falling into overfitting and reduce complex co-adaptation relationships between neurons, which is set to 0.2. And then two fully connected layers of 64 neurons are used to fuse the features of the Hi-C matrix and chromatin landscape matrix. Meanwhile, the batch-normalization layer is used to speed up the model convergence and prevent the gradient explosion and disappearance during the calculation process. Finally, the sigmoid layer is used to output the probabilities of candidate chromatin loops. A more detailed description of the framework is shown in S2 Table.

### Model training

For a fair comparison with the competing methods, we used the leave-one-out for training, validation, and testing. More specifically, 22 chromosomes were used for training and validation, where 80% of chromosomes is used for training and 20% of chromosomes is used for validation, and the remaining one chromosome is used for testing. DLoopCaller regards the prediction of genome-wide chromatin loops as a binary classification task, hence the binary cross-entropy loss (BCELoss) is used for training model, which is defined as follows:

$$BCE=-Y_{i}*\log\left(\bar{Y_{i}}\right)-(1-Y_{i})*log(1-\bar{Y_{i}})$$ (2)

where *Y_i_* denotes original values and $\bar{Y_{i}}$ denotes the predicted value of the *i*-th sample. The BCELoss is optimized by the Adam optimization algorithm [42] with a batch size is 128, and the learning rate is set to 0.001. During the training, DLoopCaller applied five-fold cross-validation to iteratively select the best parameters for distinguishing whether it is a chromatin loop and saved the model. Our proposed model is written by Python based on the Pytorch framework. We used a machine with Tesla K40 GPU with 10GB memory for training on the Linux system.

### Identifying genome-wide chromatin loops

Once the best model for each chromosome is trained, it can be used to predict all potential chromatin loops in the corresponding chromosome. Identifying chromatin loops from the whole genome includes two stages: one is to use the best trained model to score all potential chromatin loops, and the other is to pool candidate chromatin loops. Firstly, we used the best trained model to score all non-zero pixels meaning potential chromatin loops on each chromosome. Some studies have shown that the higher the interaction frequency in the Hi-C map, the greater the probability of becoming a chromatin loop [8]. Hence, to accurately and efficiently predict chromatin loops, DLoopCaller only retained those candidate chromatin loops whose contact frequency is greater than the average of all candidate chromatin loops. Finally, we used the greedy algorithm provided by Peakachu [36] to cluster all candidate chromatin loops and selected the most representative pixels as the identified chromatin loops.

## Results

### The overall performance of DLoopCaller on different cell lines

To train and measure the performance of our proposed method DLoopCaller, we performed DLoopCaller on three human cell lines (GM12878, K562 (replicate1), H1-ESC) and a mouse cell line (mESC) to validate its classification performance. The F1-score and PRAUC (Area Under the Precision-Recall Curve) metrics were employed to verify the performance of DLoopCaller and competing methods in distinguishing whether it is a chromatin loop, which were defined in S1 Note. Since the recent methods are limited to identifying a protein of interest-mediated chromatin loops [27,33], we mainly compared the proposed method DLoopCaller with a comprehensive method Peakachu in this part, and all the same enriched data were performed on both methods separately to validate the effectiveness of deep learning framework and accessible chromatin landscapes. Peakachu used the interaction frequency and rank as features in smaller matrices based on a random forest approach, outperforming Gaussian Naïve Bayes, Perceptron, Logistic Regression, SVM (linear kernel), and SVM (RBF kernel). In the GM12878 cell line, five enriched experimental data were used to label positive samples and train the model, including CTCF ChIA-PET, H3K27ac HiChIP, SMC1 HiChIP, RAD21 ChIA-PET, and promoter Capture Hi-C. The corresponding CTCF ChIA-PET in K562 (replicate1) and H1-ESC, and SMC1 HiChIP in mESC were separately used to train the model. To comprehensively evaluate the classification performance of DLoopCaller, we used the average value of F1-score, PRAUC, Precision and Recall for all chromosomes in each cell line.

**Fig 3:**
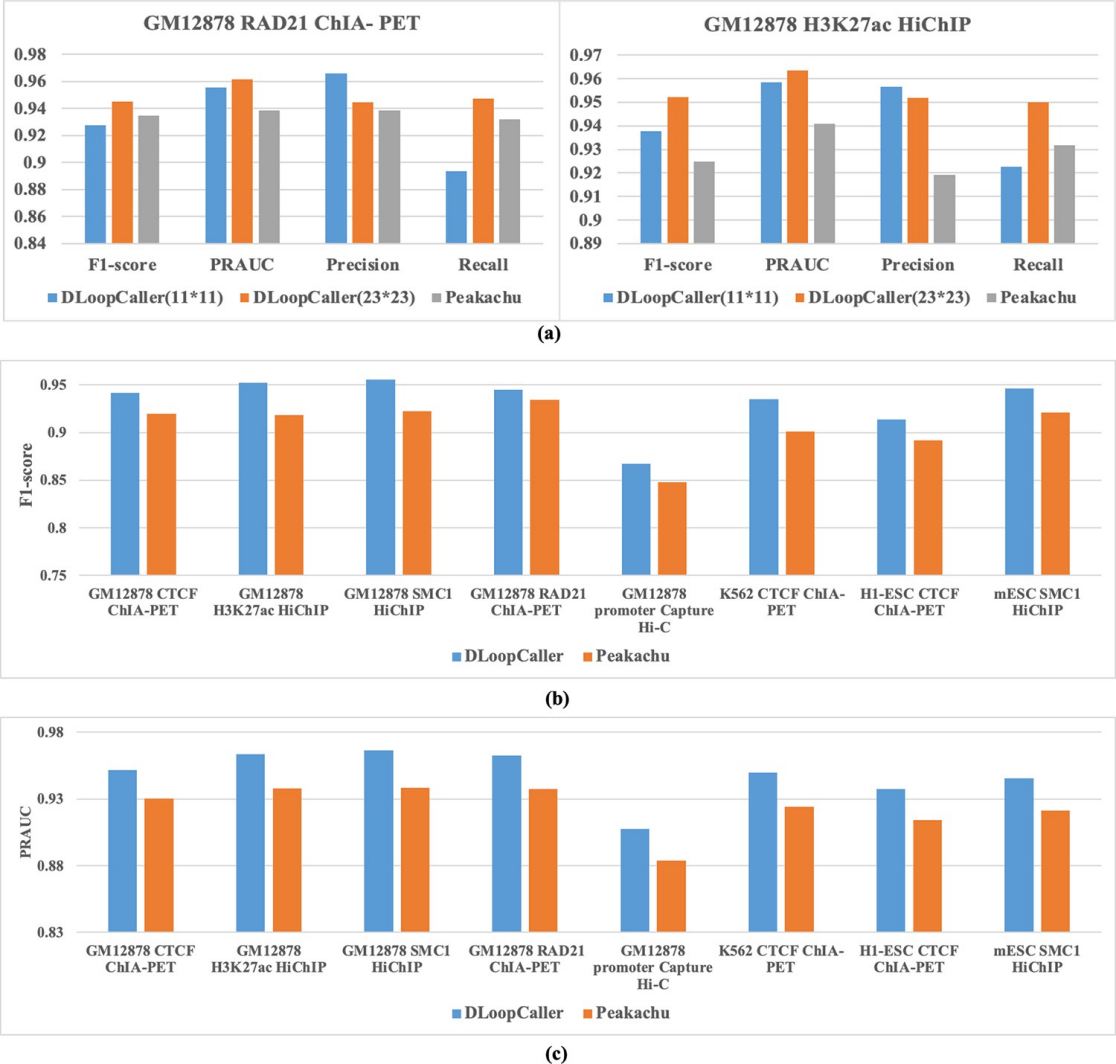
The overall performance comparison of DLoopCaller and Peakschu on different cell lines. (a) The F1-score, PRAUC, Precision and Recall values of RAD ChIA-PET and H3K27ac HiChIP in GM12878. (b)-(c): The F1-score and PRAUC values of CTCF ChIA-PET, H3K27ac HiChIP, SMC1 HiChIP, RAD ChIA-PET, and promoter Capture Hi-C in GM12878, CTCF ChIA-PET in K562 (replicate1) and H1-ESC, and SMC1 HiChIP in mESC.

Our proposed method DLoopCaller uses deep learning framework to automatically learn features instead of hand-designed features used in Peakachu for the identification of chromatin loops, which is one of the innovations of our approach. In order to fully extract the features of chromatin loops, we use a larger window (23*23) to generate the feature matrix of positive and negative samples. To better illustrate this issue, we extend DLoopCaller with window 11*11 on H3K27ac HiChIP and RAD21 ChIA-PET GM12878. As shown in Fig 3(A), we can see that even though DLoopCaller uses 11*11 window size of each interaction to generate features, the performance of it is better than peakachu overall. The experimental results confirm our assumption that DLoopCaller with a larger window (23*23) performs better than Peakachu to identify chromatin loops. The window size is only a parameter, where larger window better fit DLoopCaller to improve the identification accuracy of chromatin loops. Although Peakachu and DLoopCaller use different window size, it is relatively fair for experimental purposes. And another innovation of our method is to efficiently combine one dimensional (1D) open chromatin landscapes with 3D genomic data for chromatin loops prediction, which is also not considered by Peakachu. Therefore, a larger window size (23*23) is adopted in DLoopCaller for the following experiments.

From Fig 3, we can see that the average of both F1-score and PRAUC of DLoopCaller are greater than Peakachu, indicating that the classification performance of DLoopCaller is better than that of Peakachu on all cell lines. As shown in Fig 3(B) and 3(C), which shows the experimental results of five enriched datasets in GM12878, we can see that the average F1-score and PRAUC value in the CTCF ChIA-PET, H3K27ac HiChIP, SMC1 HiChIP, RAD21 ChIA- PET are both close to 0.95 or greater than 0.95, showing a relatively excellent classification performance. It is worth noting that the F1-score of method DLoopCaller is about 8% higher than Peakachu in K562 (replicate1), but the F1-score and PRAUC value of the two methods are relatively lower compared to other cell lines. And the line and box plots of detailed results about F1-score and PRAUC were shown in S1 and S2 Figs, the performance of DLoopCaller obviously outperformers Peakachu on most of chromosomes. According to the boxplots of precision and recall values shown in S3 Fig, DLoopCaller is better than Peakachu except the precision of DLoopCaller is slightly lower than that of Peakachu in H1-ESC. The overall experimental results show that DLoopCaller combining Hi-C contact maps with accessible chromatin data to facilitate the prediction of genome-wide chromatin loops.

### Performance assessment from different enriched experimental data within individual cell types

**Fig 4:**
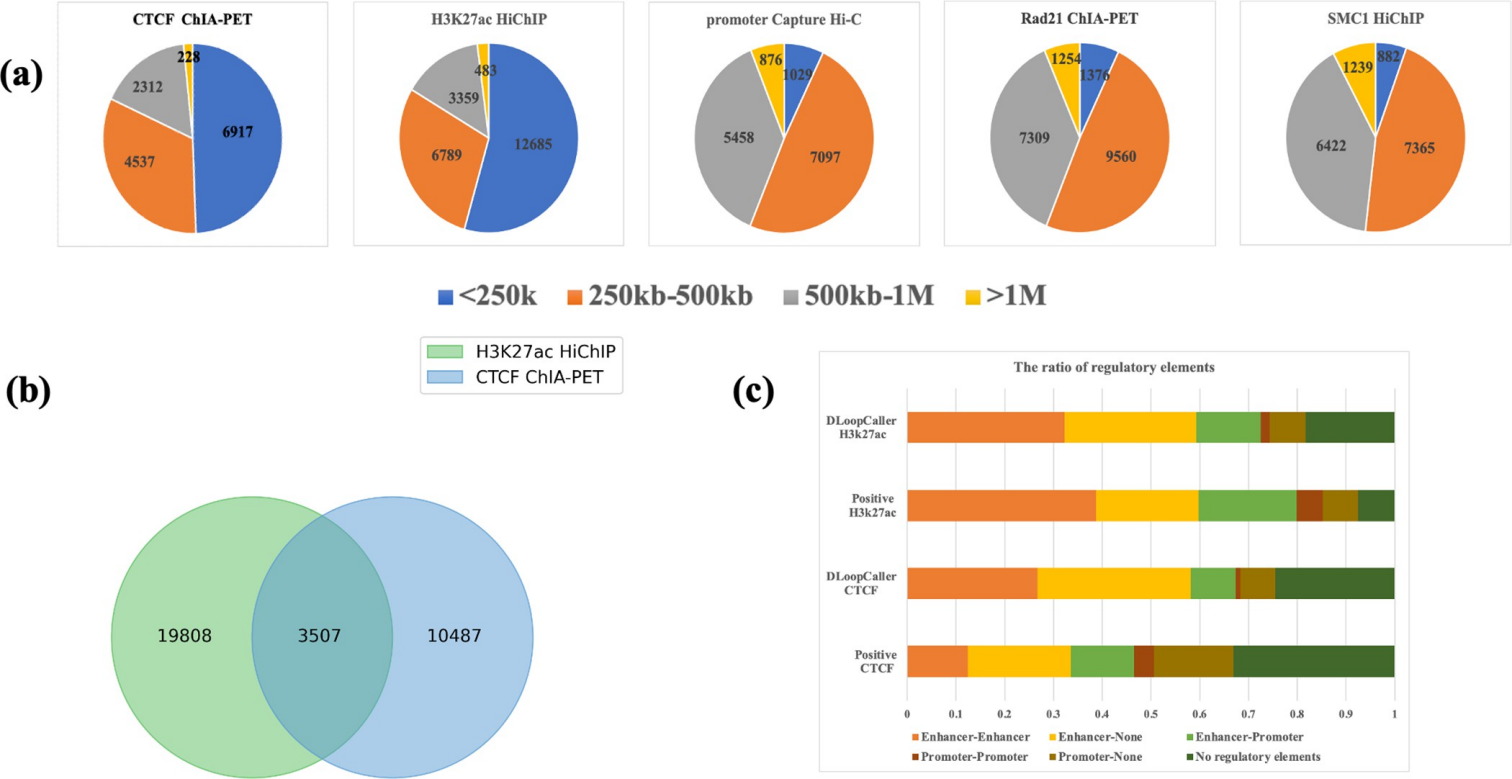
Comparison of chromatin loops within individual cell types. (a) Distance distribution of DLoopCaller identified chromatin loops from Hi-C contact maps by using CTCF ChIA-PET, H3K27ac HiChIP, SMC1 HiChIP, RAD ChIA-PET, and promoter Capture Hi-C data after training on GM12878. (b) Venn diagram of DLoopCaller identified chromatin loops determined by CTCF ChIA-PET and H3K27ac HiChIP experiments in GM12878. (c) The proportion of CTCF ChIA-PET interactions and H3K27ac HiChIP interactions types for GM12878. The proportion of identified chromatin loops types using CTCF ChIA-PET data and H3K27ac HiChIP after training for GM12878.

In order to further assess the performance of the proposed DLoopCaller, chromatin loops predicted from genome-wide contact maps were analyzed. We firstly performed experiments on CTCF ChIA-PET, H3K27ac HiChIP, SMC1 HiChIP, RAD21 ChIA-PET, and promoter Capture Hi-C in GM12878, and analyzed the differences of predicted chromatin loops within individual cell types. The best trained model of each chromosome was used to predict chromatin loops, and then we aggregated the identified chromatin loops on all chromosomes for further analysis. As shown in Fig 4(A), the distance distribution of the identified chromatin loops in different enrichment experimental data varies, for example, the distance distributions of SMC1 HiChIP, RAD21 ChIA-PET, and promoter Capture Hi-C is similar, mainly located in 250kb and 500kb, and the proportions are 46.3% (7365/15908), 48.8%(9560/19590) and 49.1%(7097/14460). While CTCF ChIA-PET and H3K27ac HiChIP loops are mainly located in less than 250kb, the latter is about 5% higher than the former. The results confirm that the distances of long-range interactions are correlated with the factor of interest when using the same sequencing method [43].

To further assess this difference, the Aggregated Peak Analysis (APA) was used to quantify how well each chromatin loop set was supported by the Hi-C signals [7]. The APA plots of chromatin loops captured by the five enriched experiments in GM12878 are shown in S4 Fig. These APA plots show considerable enrichment compared to their local background and show strong consistency in GM12878 using different enrichment experiments. As shown in Fig 4(B), the overlapping chromatin loops of CTCF ChIA-PET and H3K27ac HiChIP loops only account for a quarter, which means the two anchors completely matched of two bins, even though the distance distribution of both is similar.

Some studies have shown that H3K27ac is an active enhancer- and promoter-associated histone marker and H3K27ac HiChIP can identify functional enhancer-promoter interactions with high confidence [1,44], and CTCF ChIA-PET aims to detect the specific long-range interactions [43]. Therefore, we analyzed the proportion of regulatory elements in the H3K27ac HiChIP data and identified H3K27ac HiChIP chromatin loops in GM12878. We find that the majority of the interactions and the identified loops in H3K27ac HiChIP data are mediated by enhancers, and the ratios are very close accounting for about 80% and 75% respectively. Compared to H3K27ac HiChIP data, the interactions in CTCF ChIA-PET are relatively smaller accounting for 47%, but the interactions without regulatory elements are relatively larger accounting for 30%. And the majority of identified chromatin loops in CTCF ChIA-PET are enhancer-mediated but have more long-range interactions. These results suggest that DLoopCaller is able to predict enhancer-regulated chromatin loops with high sensitivity, which may contribute to deciphering the principles of gene expression and disease-associated genetic variants.

### Comparison of chromatin loops identified by different methods

**Fig 5:**
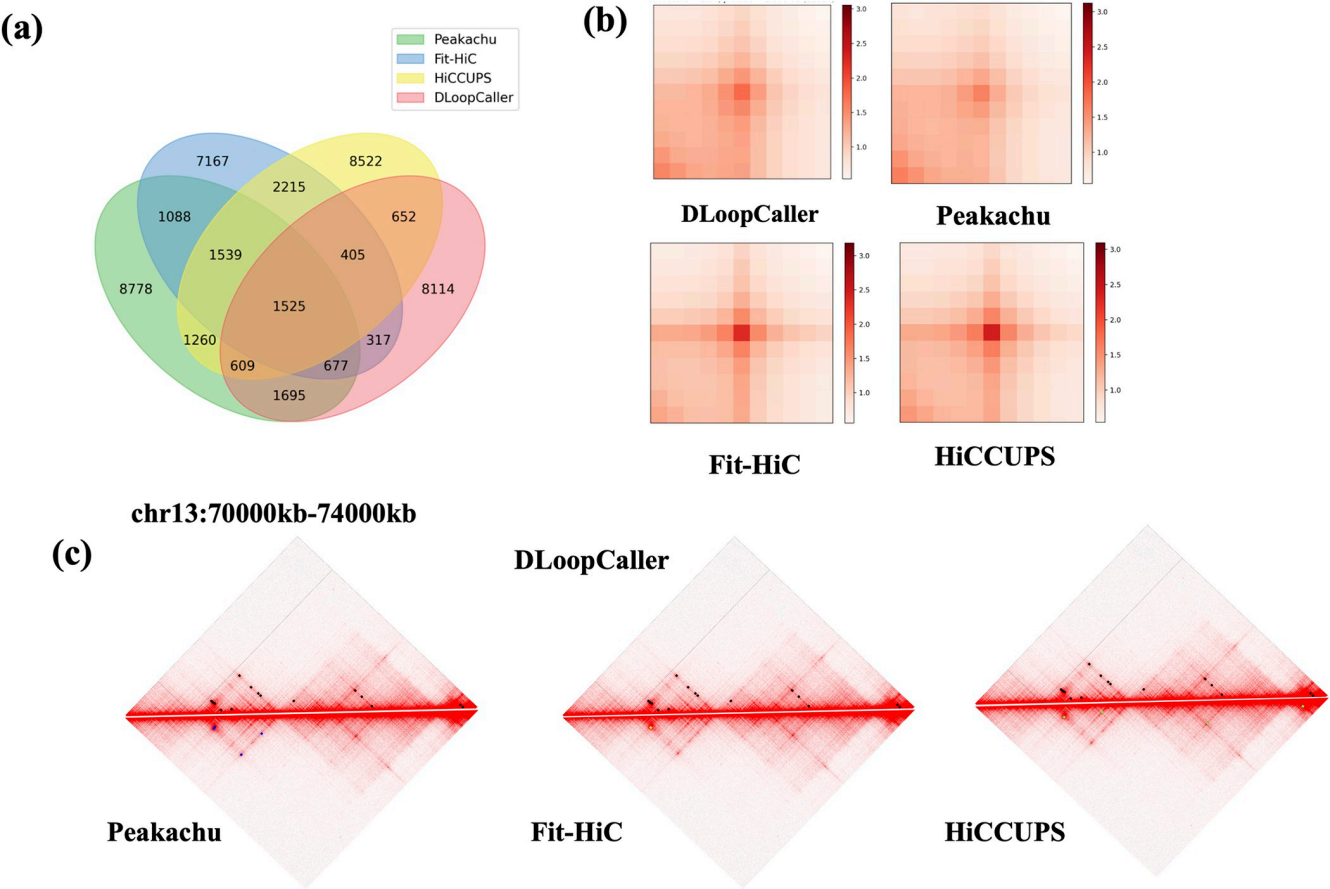
Comparison of chromatin loops identified by DLoopCaller, HiCCUPS, Fit-HiC, and Peakachu in GM12878. (a) Venn diagram of identified chromatin loops determined by DLoopCaller CTCF ChIA-PET, HiCCUPS, Fit-HiC, and Peakachu in GM12878. (b) APA plots for DLoopCaller CTCF ChIA-PET loops, HiCCUPS, Fit-HiC, and Peakachu in GM12878. (c) A visual example of identified loops by different models in a region. The black dots in the upper half of the three diamond-shaped graphs represent the chromatin loops identified by DLoopCaller, and the blue, green, and yellow dots in the lower half represent the chromatin loops identified by Peakachu, Fit-HiC, and HiCCUPS respectively.

To further validate the performance of the proposed DLoopCaller and increase the confidence of the identified chromatin loops, we compared CTCF ChIA-PET loops identified by DLoopCaller with some of the most popular methods, including Peakachu, global enrichment-based method Fit-Hi-C, and local enrichment-based methods HiCCUPS. For a fair comparison, the competing methods were also performed at 10kb resolution in GM12878 respectively and the identified chromatin loops were filtered to maintain the close number of DLoopCaller. We first compared the chromatin loops identified by each method by considering overlapping when the anchors of the two chromatin loops matched completely. As shown in Fig 5(A), we find that 42% (5880/13994) of identified chromatin loops by DLoopCaller are overlapped with ones by the other three methods, and 8114 chromatin loops are unique. We specifically compared CTCF ChIA-PET chromatin loops and H3K27ac HiChIP chromatin loops respectively identified by DLoopCaller and Peakachu. As shown in S5(A) and S5(B) Fig, the number of CTCF ChIA-PET loops of Peakachu and DLoopCaller is basically the same with the overlapping ratio 29.3%(4105/13994), and DLoopCaller identifies more H3K27ac HiChIP loops than Peakachu with the overlapping ratio 18.17% (4236/23315). From the perspective of the distance distribution of the identified chromatin loops, the distance distributions of the H3k27ac HiChIP loops identified by the two methods are very similar, but the proportion of long-range (>250kb) CTCF ChIA-PET chromatin loops identified by DLoopCaller is slightly lower than that of Peakachu (S5(C) and S5(D) Fig). The APA plots are used to inspect the overall loop patterns of the detected peaks by all methods, the APA plots of Fit-HiC and HiCCUPS show strong consistency mainly focusing on the center pixel. Overall, the APA plots of DLoopCaller and Peakachu show similar enrichment of contact signals compared to surrounding pixels, but the former has a slightly stronger enrichment signal concentrating on the center pixel than the latter.

Taken together, the genome-wide analysis described above demonstrates that DLoopCaller has a good capability in terms of the identified loops from HiC contact maps. To further illustrate this point, we used juicebox (https://github.com/aidenlab/Juicebox/wiki), a visualization tool embedded in the juicer tool [24], to visualize some examples of the identified loops. We can see from Fig 5(C) that most of the chromatin loops identified by DLoopCaller and other methods in this region are overlapped but some are unique. And more visual examples are shown in S6 Fig.

### Chromatin loops reveal cell-type specificity

**Fig 6:**
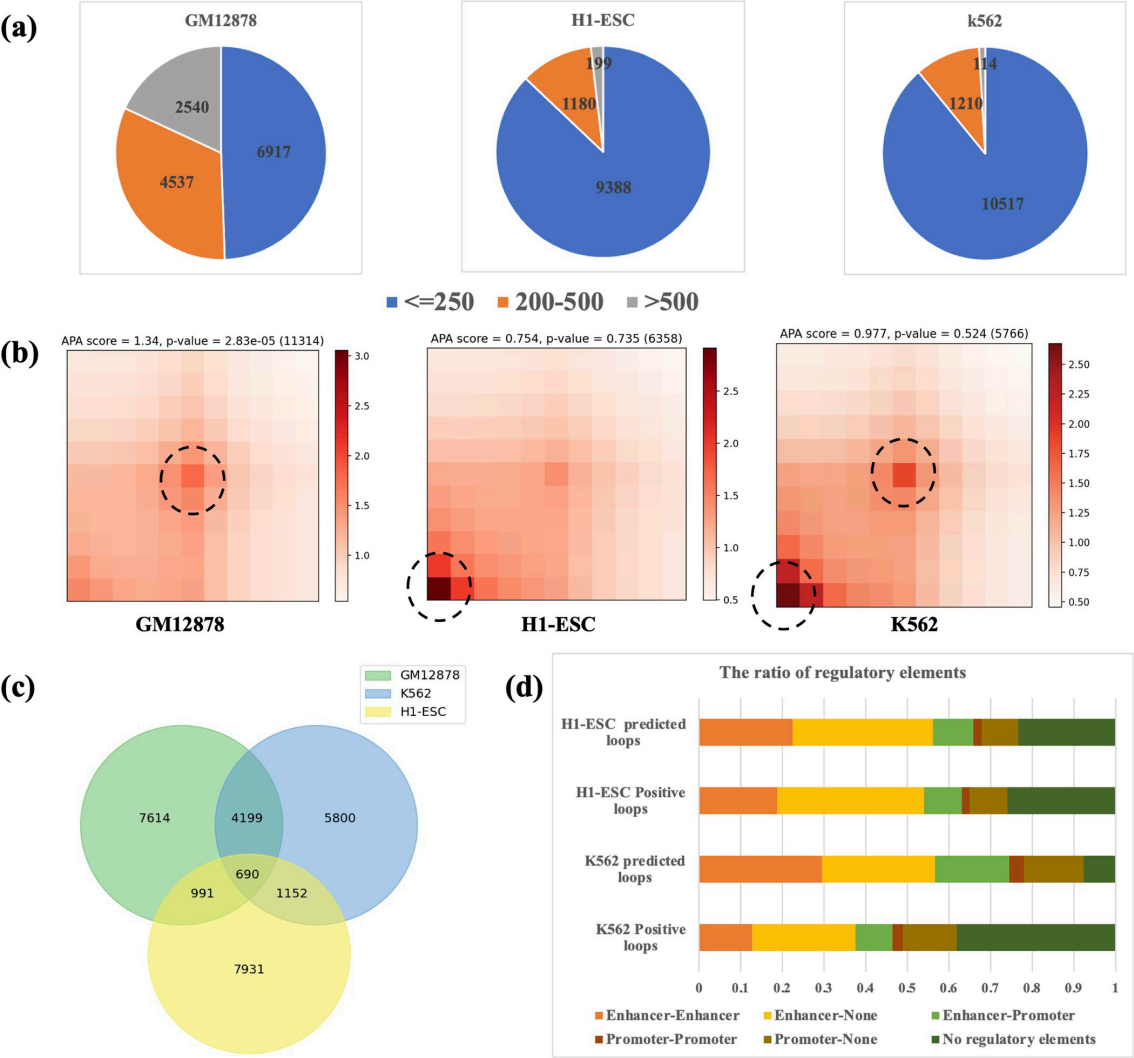
Comparison of chromatin loops among cell types. (a) Distance distribution of DLoopCaller identified chromatin loops from Hi-C contact maps by using CTCF ChIA-PET data after training on GM12878, H1-ESC, and K562 (replicate1) separately. (b) APA plots of identified chromatin loops in GM12878, H1-ESC, and K562 (replicate1). (c) Venn diagram of DLoopCaller identified chromatin loops determined by CTCF ChIA-PET experiments in GM12878, H1-ESC and K562 (replicate1). (d) The proportion of CTCF ChIA-PET interactions types for H1-ESC and K562. The proportion of identified chromatin loops types using CTCF ChIA-PET data after training for H1-ESC and K562 (replicate1).

Next, we evaluated the ability to identify loops in other cell lines. DLoopCaller separately identified 13994 CTCF ChIA-PET loops in GM12878, 10767 SMC1 HiChIP loops in H1-ESC, and 11841 CTCF ChIA-PET loops in K562 (replicate1), of which the short-range (< 250kb) interactions account for 49.4%, 87.2%, and 88.8%, respectively. To further illustrate the differences, we analyzed the APA profiles of all identified chromatin loops in three cell lines as shown in Fig 6(B). We find that the most important predictor is the center pixel and bottom left pixel respectively in GM12878 and H1-ESC, while it is jointly driven by the center and bottom left pixel in K562 (replicate1). In addition, we compared the overlap of chromatin loops in the three cell lines, and any anchors in the two bins are allowed to be incompletely matched to increase the fault tolerance. Briefly, two chromatin loops were considered matched if the ±10kb region around the center of one loop overlaps another. The comparison results are shown in Fig 6(C), even the tolerance for overlapping is increased, the overlapping chromatin loops of the three cell lines are also relatively less, which proves that the identified chromatin loops are cell-type specific.

In addition, to further analyze the relationship between the chromatin loops identified by DLoopCaller and the regulatory elements in all cell lines. As shown in Fig 6(D), the proportion of each regulatory element of SMC1 HiChIP data and identified chromatin loops in H1-ESC is very similar, which indicates that the chromatin loops identified by the DLoopCaller are reliable and demonstrates that the proposed DLoopCaller is effective. From Figs 4(C) and 6(D), we can conclude that the ratio of the regulatory elements in the chromatin loops identified by DLoopCaller and training data is basically the similar, and most of chromatin loops are regulated by enhancers. This experimental result has also been verified by the existing research [45], which provides the possibility to further understand the gene regulatory network. We also found that these predicted cell-type-specific loops are often located chromatin open regions and active enhancer regions (S7 Fig).

### Transcription factor motif co-enrichment across different cell lines and species

**Fig 7:**
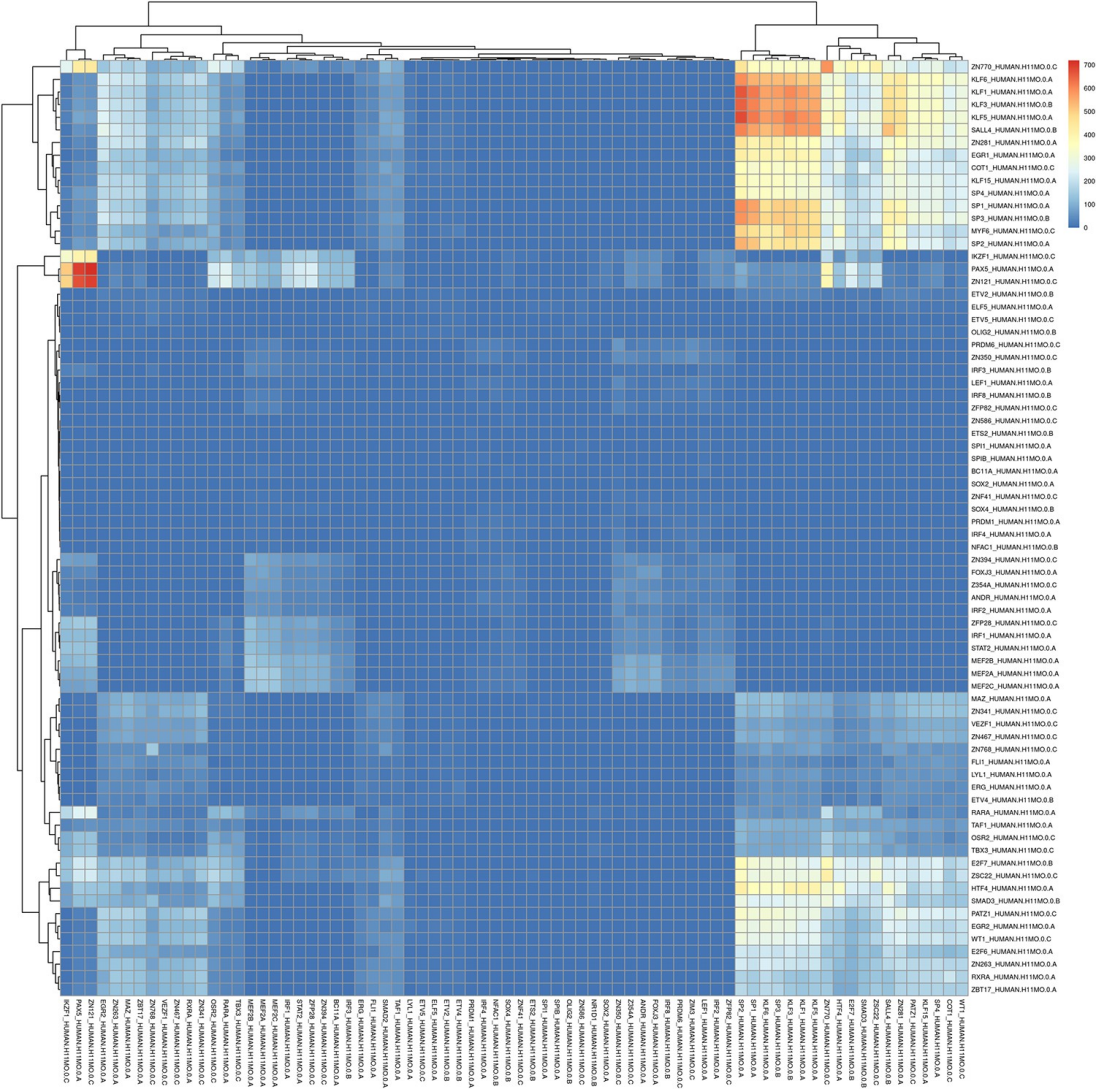
The co-enrichment of transcription factors on identified chromatin loops in H1-ESC with CTCF ChIA-PET training model.

Some studies have demonstrated that enhancer-promoter interactions regulate target genes in the genome, and specific transcription factor cooperation offers the possibility to understand the cell-type specificity of genome interactions [46], of which Cicero, PEP, and the latest proposed Spatzie attempted to detect the transcription factor motif cooperativity between enhancer-promoter interactions [47–49]. To analyze whether the sequence-based features within identified chromatin loops, we first performed experiments using Spatzie where all identified chromatin loops were used in each cell line. We applied Spatzie with count correlations to estimate cooperativity and showed the strongest enrichment between KLF5 and ZN700, KLF3, KLF6, SP2, and SP1 motifs in H1-ESC (Fig 7). Moreover, the cooperativity estimations in GM12878 and K562 (replicate1) were shown in S8 and S9 Figs, it is obvious that the strongest enrichment between ZN770 and PAX5, and ZN121 motifs, between IKZF1 and PAX5 in K562, strongest enrichment between ZN700 and E2F7, and ZSC22 motifs in GM12878. This phenomenon suggests that motif enrichment differs in the identified chromatin loops from different cell lines, which may provide helpful analysis of transcriptional regulation.

In addition, we find that the majority of the SMC1 HiChIP loops in mESC are distributed within 250kb accounting for up to 90.4% (13288/14695), and the APA plots show that the enrichment is more obvious in the lower left, which is similar to H1-ESC (S10(a)-(b) Fig). As shown in S6(C) Fig, and the overlap ratio of SMC1 HiChIP loops in mESC and GM12878 is 13.2% (2424/18332) even mismatches between either anchor are allowed, which suggests that the identified chromatin loops are specific across species even using the same enrichment technology. Moreover, from S11 and S12 Figs, the transcription factor motif co-enrichment in GM12878 and mESC demonstrates specificity. We conclude that the identified chromatin loops exhibit specificity for significant transcription factor motif co-enrichment across different cell lines and species.

### The reproducibility and robustness of DLoopCaller

We also evaluated the degree of repeatability and robustness of DLoopCaller across biological replicates and differennt sequencing depth. We performed DLoopCaller on two replicates of K562 with CTCF ChIA-PET training to verify the reproducibility of DLoopCaller. Due to the large difference in mapping read size between the two replicates, we used different thresholds to keep the similar number of chromatin loops identified in the two replicates. As shown in S13 Fig, with regard to the distance distribution, APA analysis, and the proportion of regulatory elements of identified chromatin loops exhibit similarity in the two replicates. And it is obvious that the common strongest enrichment between ZN770 and PAX5, and ZN121 motifs in the two replicates (S8 and S14 Figs). Overall, the chromatin loops identified by DLoopCaller in the two replicates are similar to some extent, which proves that DLoopCaller is reproducible. In addition, we adopted a binomial probability used in Peakachu to downsample the contact map of GM12878 without re-mapping, and we performed DLoopCaller with H3K27ac HiChIP training on the 80%,50% and 30% down-sampled matrix respectively with 1.6 billion, 1 billion and 600 million cis-reads. The experimental results show that the identified loops at different sequencing depths maintain a large degree of overlap with those on original hic matrix, especially 77.1%, 72.2% and 76.4% in the 80%,50% and 30% down-sampled matrix **(**S15 Fig**)**, which indicates DLoopCaller is robust.

## Discussion

With the rapid development of chromatin conformation capture technologies, which provides opportunities to dissect the role of the 3D structure of chromatin in cellular processes, including regulation of gene expression and DNA replication. Here, we proposed a novel method DLoopCaller integrating Hi-C contact maps and accessible chromatin landscape data to identify genome-wide chromatin loops. The main contribution of DLoopCaller lies in the following points: (i) We used the chromatin landscape data to generate a chromatin landscape matrix that matches the Hi-C contact maps, avoiding manual feature extraction; (ii) applied massive enriched experimental data, such as ChIA-PET/HiChIP and Capture Hi-C, to annotate positive samples; (iii) developed a deep learning framework to simultaneously extract features from Hi-C matrix and accessible chromatin landscape matrix to improve the accuracy of identifying chromatin loops in the whole genome. The experimental results show that DLoopCaller can effectively improve the accuracy of identifying chromatin loops compared with competing methods and identify a series of unique chromatin loops. We find that the identified chromatin loops from H3K27ac HiChIP contain more short-range loops while the ones from promoter Capture Hi-C contain more long-range loops in GM12878. Moreover, we discovery that most of the chromatin loops identified by DLoopCaller are mediated by enhancers, which is largely consistent with the used enrichment experimental data. Next, according to the analysis of the experimental results, identified chromatin loops show cell type specificity with low overlapping ratio across cell lines. Then significant transcription factor motif co-enrichment in identified chromatin loops exhibits specificity across different cell lines and across species. Last but not least, DLoopCaller is reproducible and robust across different biological replicates and sequencing depths.

Although DLoopCaller has achieves excellent performance and makes some new discoveries, there are still many limitations: (i) The training data of DLoopCaller depends on the enrichment experimental data, but the sources of different batches will produce noise in the experimental results; (ii) although DLoopCaller identifies many unique chromatin loops, they may have false positives; (ii) the adopted deep learning framework is a black-box model, which is difficult to interpret the extracted features for the identification of chromatin loops. Despite the above limitations, there are still a lot of works to be worth pursuing: (i) We perform DLoopCaller on more Hi-C data obtained from different chromatin conformation capture technologies to verify the effectiveness of the method, such as DNA SPRITE data and Micro-C maps; (ii) The results obtained by our method may be used to predict enhancer-promoter interactions to help further understanding of transcriptional regulatory mechanisms, which remains a major challenge; (iii) to date, there are some pioneering works to enhance the resolution of Hi- C data, such as HiCPlus [50], HiCNN [51], and DeepHiC [52], we could try to perform DLoopCaller on high resolution Hi-C matrices to reduce false positives; (iv) we could try to incorporate more one-dimensional chromatin maps, such as histone modification data and gene expression data, to further improve the accuracy of identifying chromatin loops; (v) improving the efficiency of predicting chromatin loops on the whole genome.

## Supporting information

S1 Note The definition of evaluation metrics. (DOCX)

S1 Table The number of samples in each dataset. (DOCX)

S2 Table The detailed settings of DLoopCaller. (DOCX)

S1 Fig The line charts about F1-score and PRAUC for all chromosomes in GM12878, K562 (replicate1), H1-ESC and mESC. (TIF)

S2 Fig The boxplots about F1-score and PRAUC for all chromosomes in GM12878, K562 (replicate1), H1-ESC and mESC. (TIF)

S3 Fig The boxplot about Precision and Recall for all chromosomes in GM12878, K562 (replicate1), H1-ESC and mESC. (TIF)

S4 Fig APA plots for CTCF ChIA-PET, H3K27ac HiChIP, SMC1 HiChIP, RAD ChIA- PET, and promoter Capture Hi-C loops in GM12878 cell lines. (TIF)

S5 Fig (a) Venn diagram of CTCF ChIA-PET chromatin loops determined by DLoopCaller and Peakachu in GM12878; (b) Venn diagram of H3k27ac HiChiP chromatin loops determined by DLoopCaller and Peakachu in GM12878; (c) Distance distribution of Peakachu identified chromatin loops from Hi-C contact maps by using CTCF ChIA-PET data after training on GM12878; (d) Distance distribution of Peakachu identified chromatin loops from Hi-C contact maps by using H3k27ac HiChiP data after training on GM12878.
(TIF)

S6 Fig Visual examples of identified loops by different models in a region. The black dots in the upper half of the three diamond-shaped graphs represent the chromatin loops identified by DLoopCaller, and the blue, green, and yellow dots in the lower half represent the chromatin loops identified by Peakachu, Fit-HiC, and HiCCUPS respectively.
(TIF)

S7 Fig The cell type-specific loops with unique cell type specific chromatin accessibility or histone modification features. (TIF)

S8 Fig The co-enrichment of transcription factor on identified chromatin loops in K562(replicate1) with CTCF ChIA-PET training model. (TIF)

S9 Fig The co-enrichment of transcription factor on identified chromatin loops in GM12878 with CTCF ChIA-PET training model. (TIF)

S10 Fig (a) Distance distribution of DLoopCaller identified SMC1 HiChIP chromatin loops from Hi-C contact maps in mESC. (b) The APA plots for SMC1 HiChIP chromatin loops in mESC. (c) Venn diagram of DLoopCaller identified SMC1 HiChIP chromatin loops in GM12878 and mESC.
(TIF)

S11 Fig The co-enrichment of transcription factor on identified chromatin loops in GM12878 with SMC1 HiChIP training model. (TIF)

S12 Fig The co-enrichment of transcription factor on identified chromatin loops in mESC with SMC1 HiChIP training model. (TIF)

S13 Fig (a) Distance distribution of DLoopCaller identified chromatin loops from Hi-C contact maps by using CTCF ChIA-PET data after training on two replicates of K562; (b) APA plots for DLoopCaller CTCF ChIA-PET loops in two replicates of K562; (c) The proportion of identified chromatin loops types using CTCF ChIA-PET data after training for two replicates of K562; (d) Venn diagram of CTCF ChIA-PET chromatin loops in two replicates of K562.
(TIF)

S14 Fig The co-enrichment of transcription factor on identified chromatin loops in K562(replicate2) with CTCF ChIA-PET training model. (TIF)

S15 Fig Concordance of identified loops from datasets different down-sampled rates. (TIF)
