# Supplementary material for: DLoopCaller: A deep learning approach for predicting genome-wide chromatin loops by integrating accessible chromatin landscapes
Source: PLoS Comput Biol. 2022 Oct 7;18(10):e1010572. doi: 10.1371/journal.pcbi.1010572 (PMC9581407; doi:10.1371/journal.pcbi.1010572)
Supplement: S1 Note — (DOCX) [file pcbi.1010572.s001.docx]

**S1 Note.** The definition of evaluation metrics.

The evaluation metrics are defined as follows：

$$\begin{aligned} F1-score= \frac{2*Precision*Recall}{Precision+Recall} \\ Precision = \frac{TP}{TP+FP} \\ Recall = \frac{TP}{TP+FN} \end{aligned}$$

where TP, FP and FN mean true positive, false positive and false negative respectively.
