# Supplementary material for: DLoopCaller: A deep learning approach for predicting genome-wide chromatin loops by integrating accessible chromatin landscapes
Source: PLoS Comput Biol. 2022 Oct 7;18(10):e1010572. doi: 10.1371/journal.pcbi.1010572 (PMC9581407; doi:10.1371/journal.pcbi.1010572)
Supplement: S1 Table — (DOCX) [file pcbi.1010572.s002.docx]

**S1 Table**. The number of samples in each dataset

| Dataset | The number of samples | Dataset | The number of dataset |
| --- | --- | --- | --- |
| GM12878 CTCF ChIA-PET | 123962 | GM12878 SMC1 HiChIP | 18987 |
| GM12878 H3k27ac HiChIP | 11880 | K562 CTCF ChIA-PET | 36974 |
| GM12878 RAD21 HiChIP | 28003 | H1-ESC CTCF ChIA-PET | 41369 |
| GM12878 promoter Capture Hi-C | 118988 | mESC SMC1 HiChIP | 17236 |
