# Supplementary material for: DLoopCaller: A deep learning approach for predicting genome-wide chromatin loops by integrating accessible chromatin landscapes
Source: PLoS Comput Biol. 2022 Oct 7;18(10):e1010572. doi: 10.1371/journal.pcbi.1010572 (PMC9581407; doi:10.1371/journal.pcbi.1010572)
Supplement: S2 Table — (DOCX) [file pcbi.1010572.s003.docx]

**S2 Table.** The detailed settings of DLoopCaller

| Architectures | Settings | Output shape |
| --- | --- | --- |
| Convolutional layer | kernel number = 16, kernel size = 5, stride = 1, padding = 2 | (*B*, *n*, 16) |
| ReLU layer | ----- | (*B*, *n*, 16) |
| Average-pooling layer | global, stride = 2 | (*B*, 16) |
| Convolutional layer | kernel number = 32, kernel size = 3, stride = 1, padding = 1 | (*B*, *n*, 32) |
| ReLU layer | ----- | (*B*, *n*, 32) |
| Average-pooling layer | global, stride = 2 | (*B*, 32) |
| Convolutional layer | kernel number = 16, kernel size = 3, stride = 1, padding = 1 | (*B*, *n*, 32) |
| ReLU layer | ----- | (*B*, *n*, 32) |
| Average-pooling layer | global, stride = 2 | (*B*, 32) |
| Batch-normalization layer | ----- | (*B*, 32) |
| Fully connected layer | unit number = 64 | (*B*, 64) |
| ReLU layer | ----- | (*B*, 32) |
| Dropout layer | 0.2 | (*B*, 32) |
| Fully connected layer | unit number = 1 | (*B*, 1) |
