# Supplementary figures and images for: DLoopCaller: A deep learning approach for predicting genome-wide chromatin loops by integrating accessible chromatin landscapes
Source: PLoS Comput Biol. 2022 Oct 7;18(10):e1010572. doi: 10.1371/journal.pcbi.1010572 (PMC9581407; doi:10.1371/journal.pcbi.1010572)

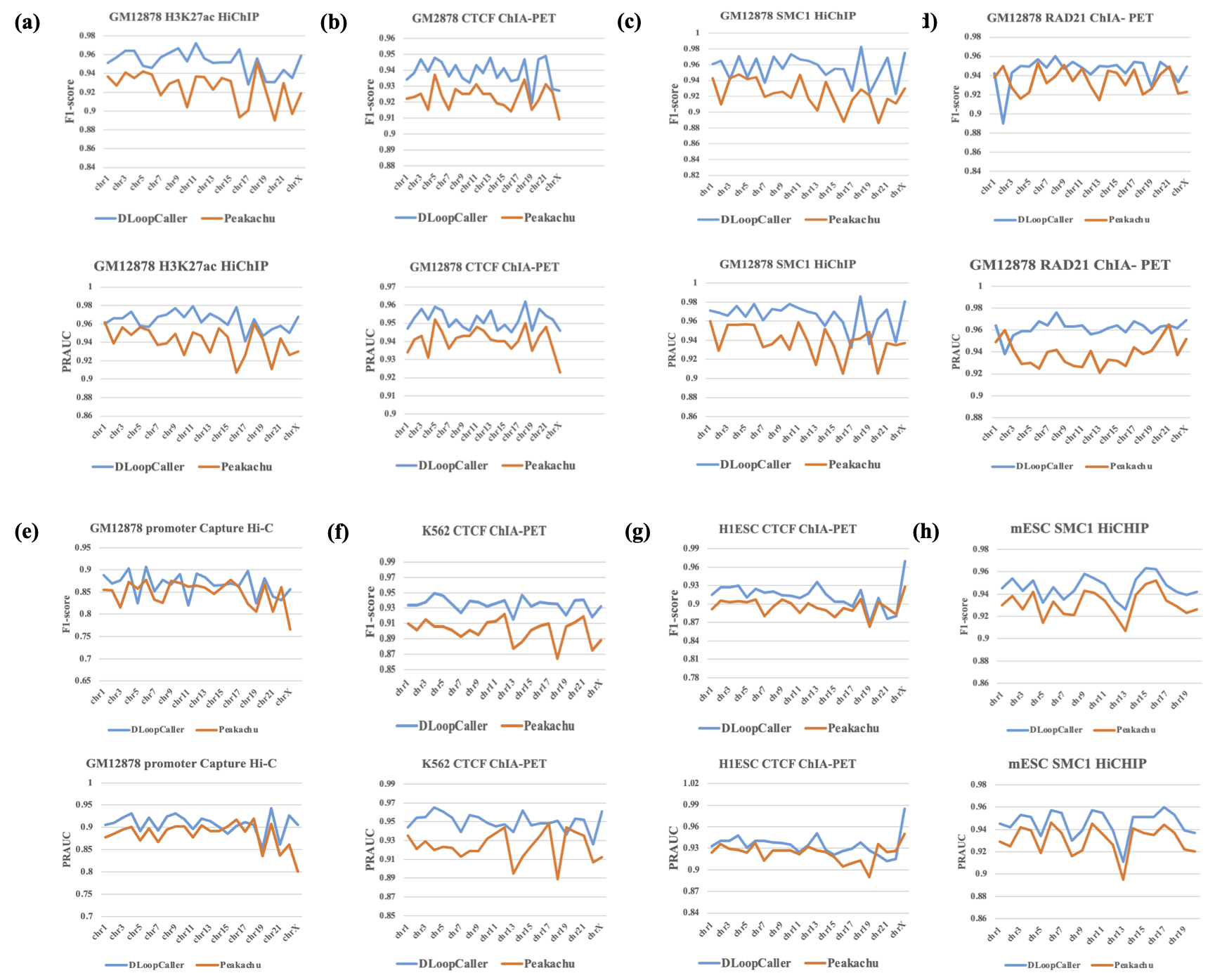

Supplement: S1 Fig — (TIF) [file pcbi.1010572.s004.tif]

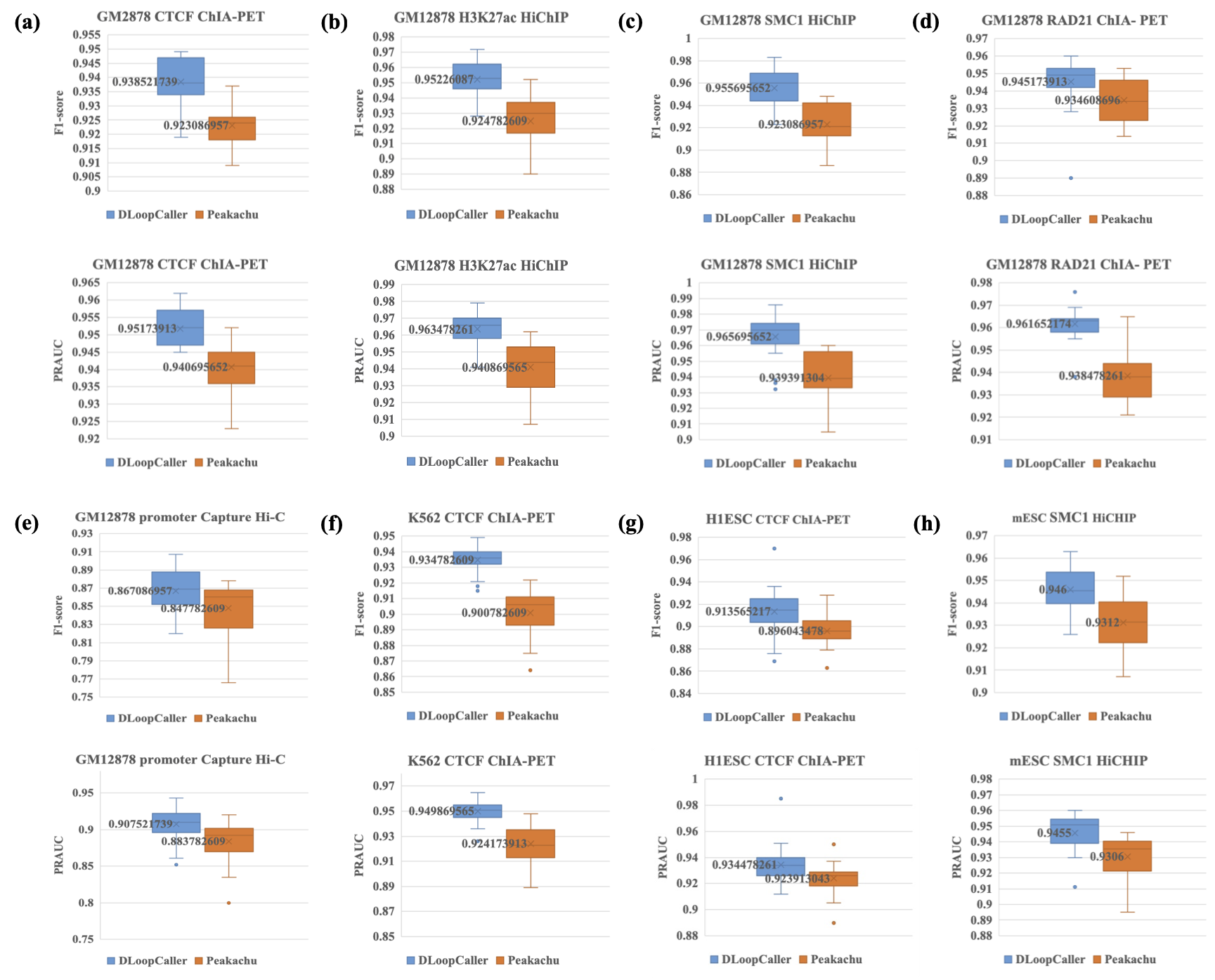

Supplement: S2 Fig — (TIF) [file pcbi.1010572.s005.tif]

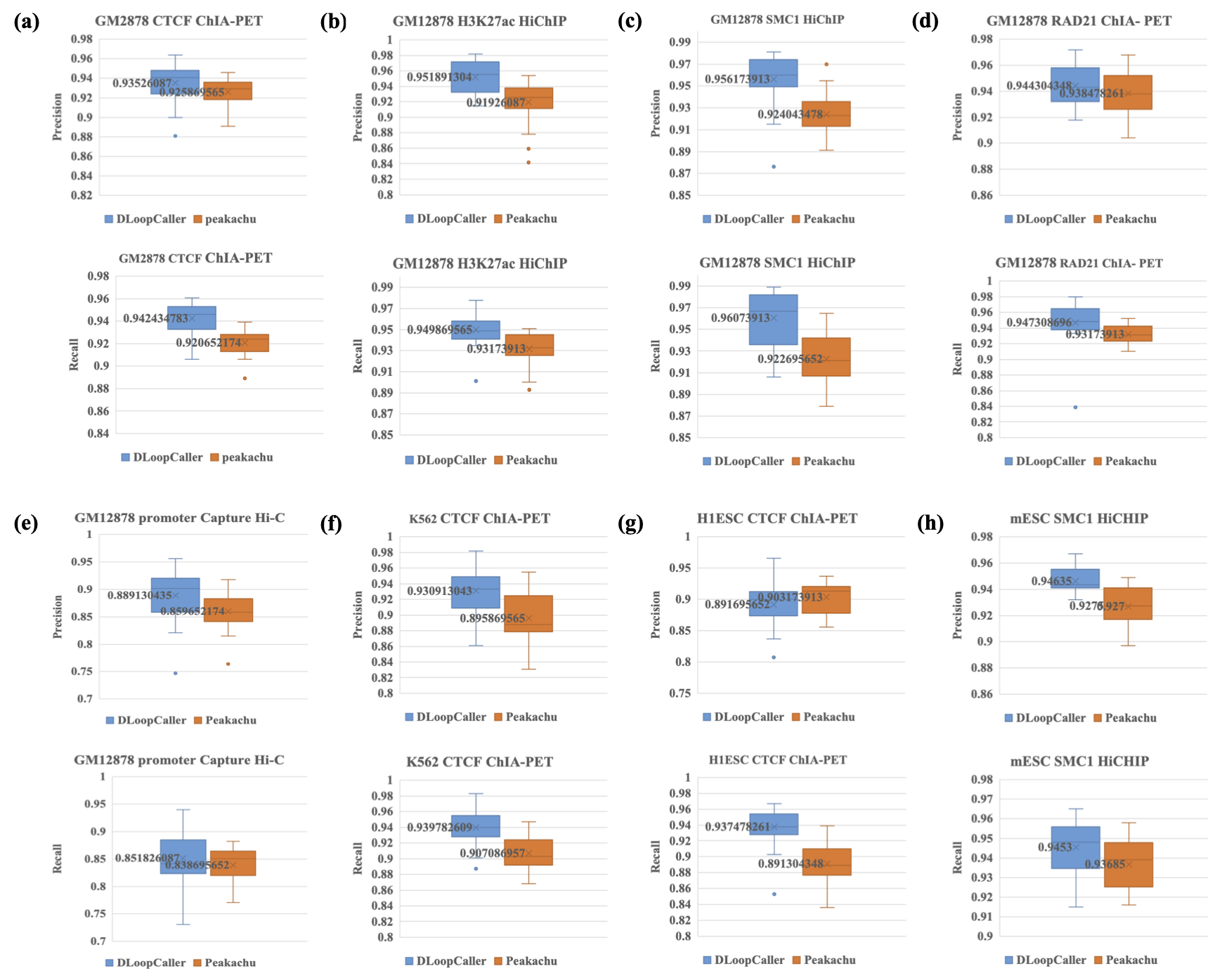

Supplement: S3 Fig — (TIF) [file pcbi.1010572.s006.tif]

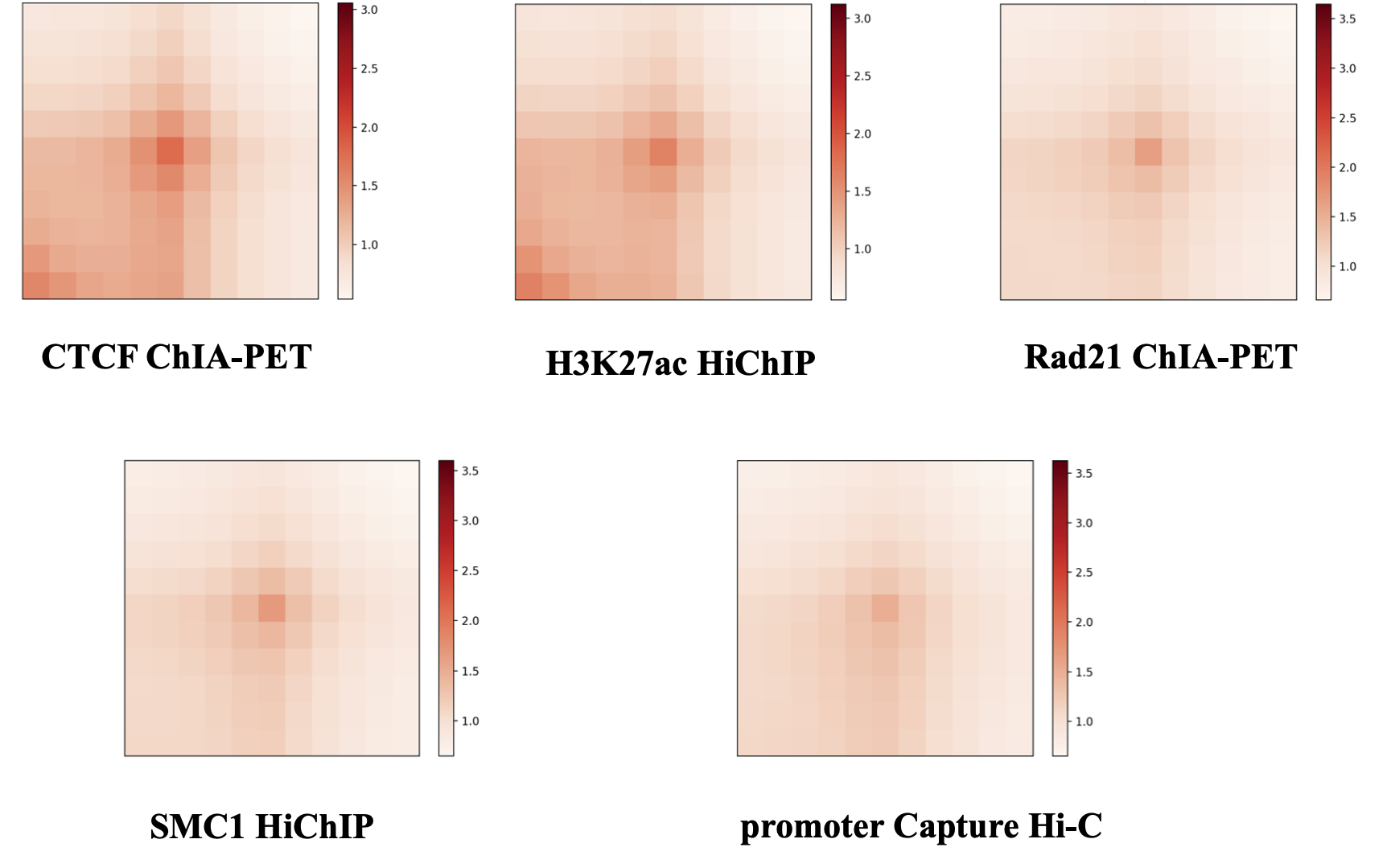

Supplement: S4 Fig — (TIF) [file pcbi.1010572.s007.tif]

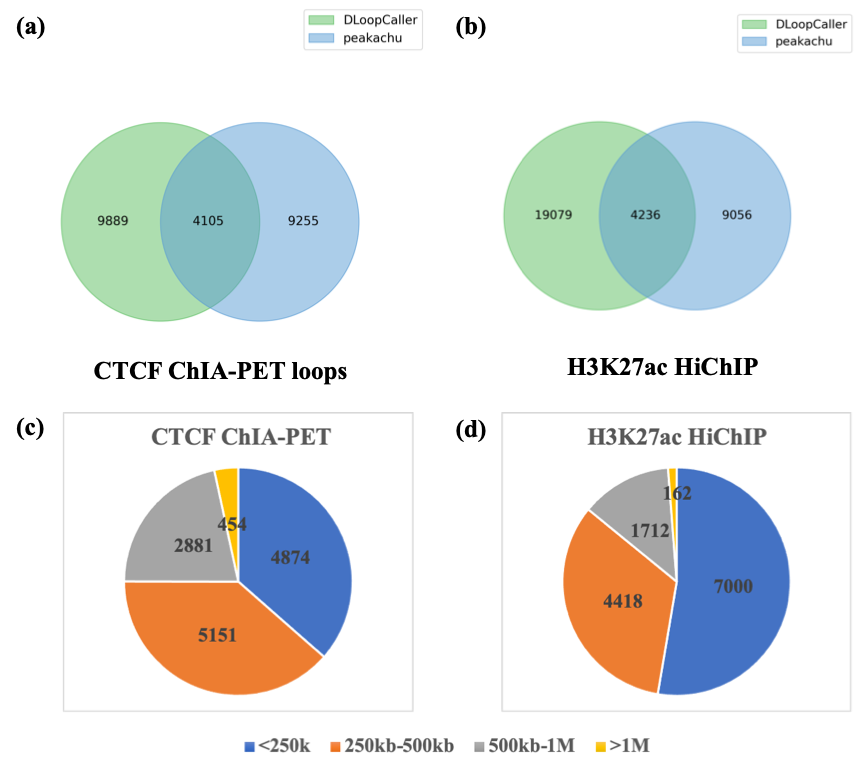

Supplement: S5 Fig — (a) Venn diagram of CTCF ChIA-PET chromatin loops determined by DLoopCaller and Peakachu in GM12878; (b) Venn diagram of H3k27ac HiChiP chromatin loops determined by DLoopCaller and Peakachu in GM12878; (c) Distance distribution of Peakachu identified chromatin loops from Hi-C contact maps by using CTCF ChIA-PET data after training on GM12878; (d) Distance distribution of Peakachu identified chromatin loops from Hi-C contact maps by using H3k27ac HiChiP data after training on GM12878. (TIF) [file pcbi.1010572.s008.tif]

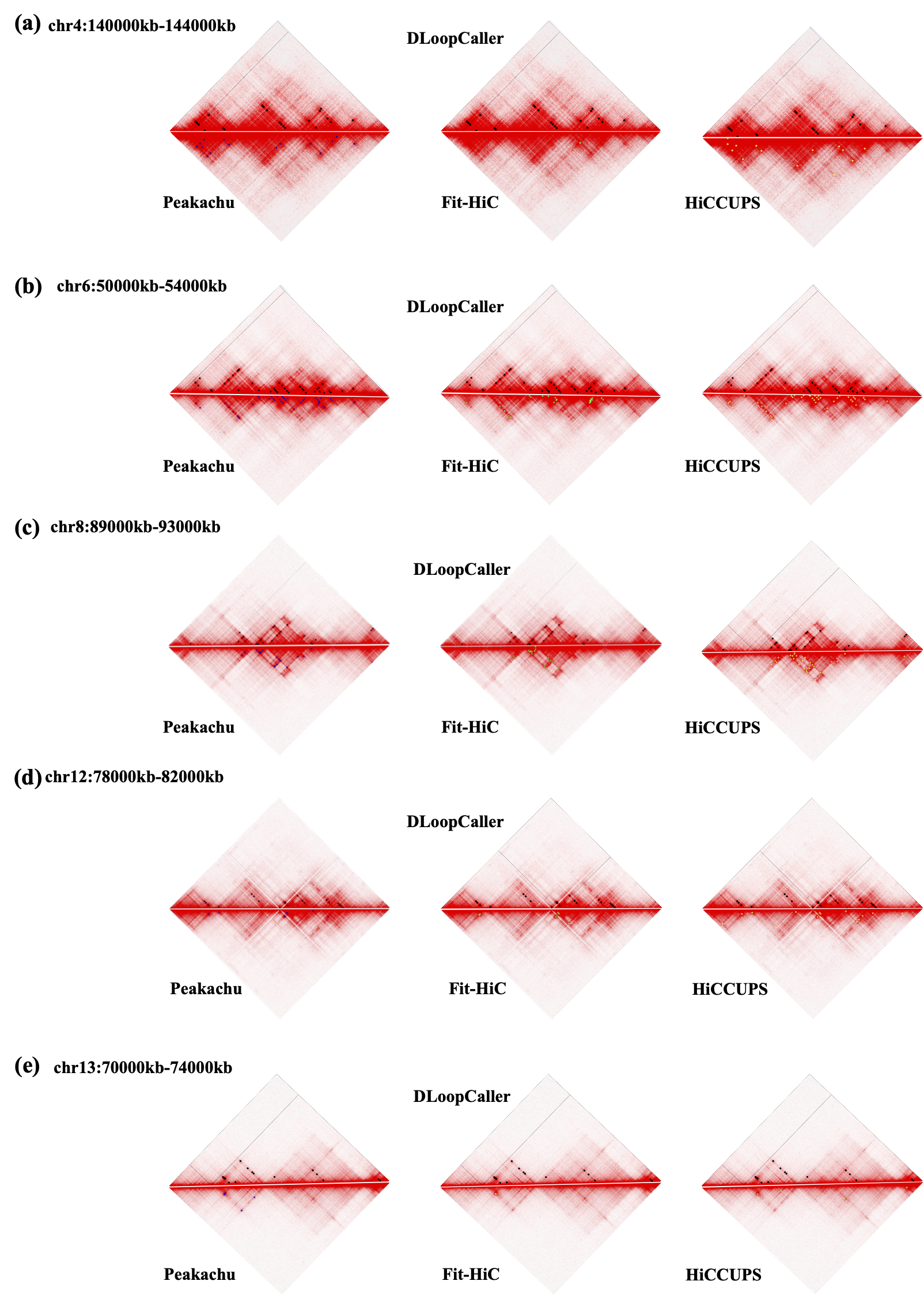

Supplement: S6 Fig — The black dots in the upper half of the three diamond-shaped graphs represent the chromatin loops identified by DLoopCaller, and the blue, green, and yellow dots in the lower half represent the chromatin loops identified by Peakachu, Fit-HiC, and HiCCUPS respectively. (TIF) [file pcbi.1010572.s009.tif]

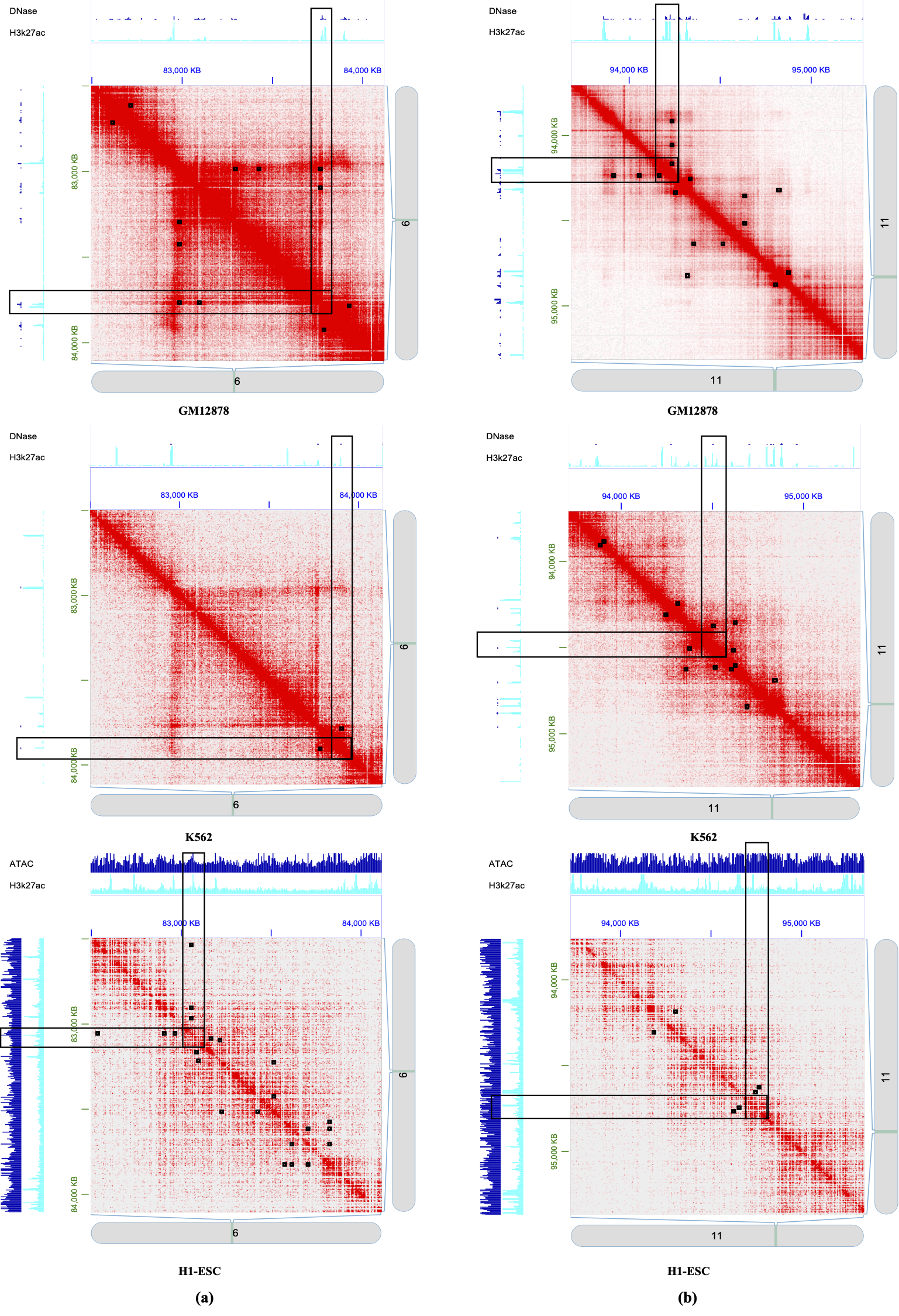

Supplement: S7 Fig — (TIF) [file pcbi.1010572.s010.tif]

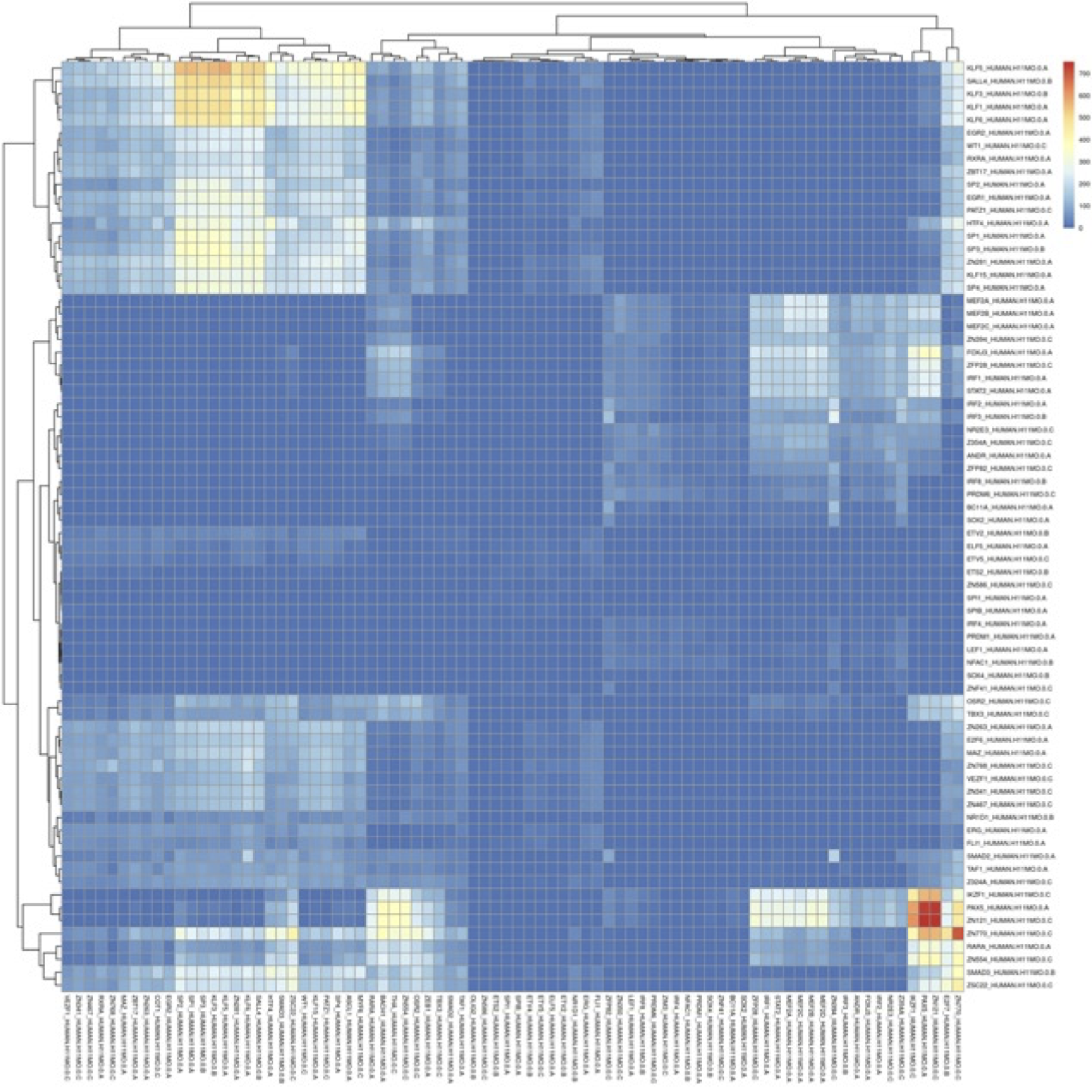

Supplement: S8 Fig — (TIF) [file pcbi.1010572.s011.tif]

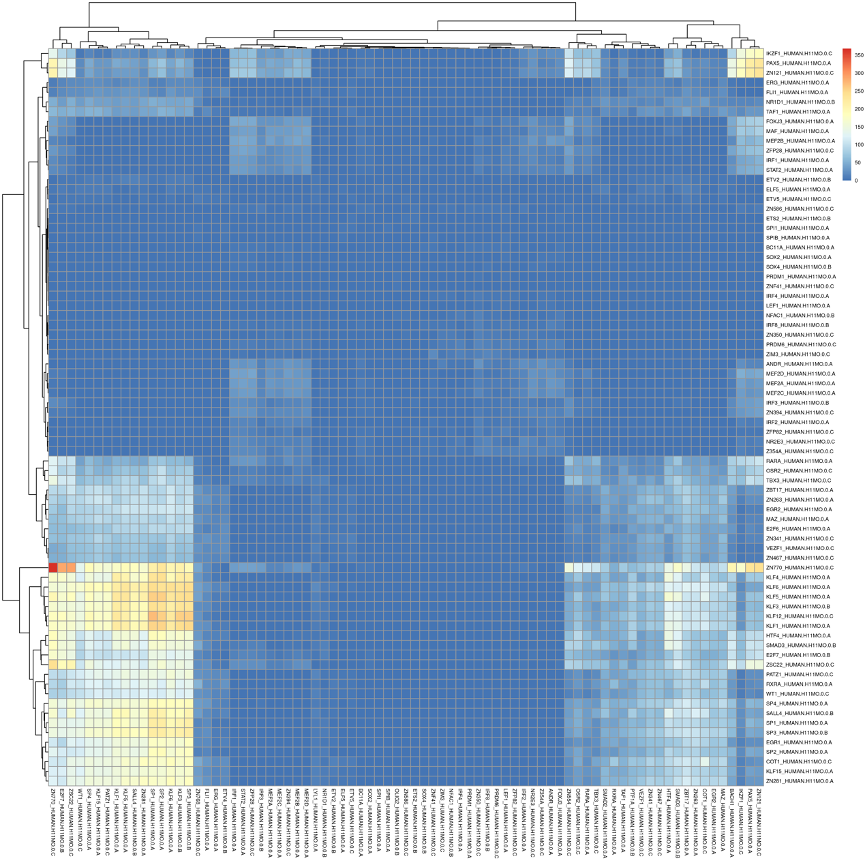

Supplement: S9 Fig — (TIF) [file pcbi.1010572.s012.tif]

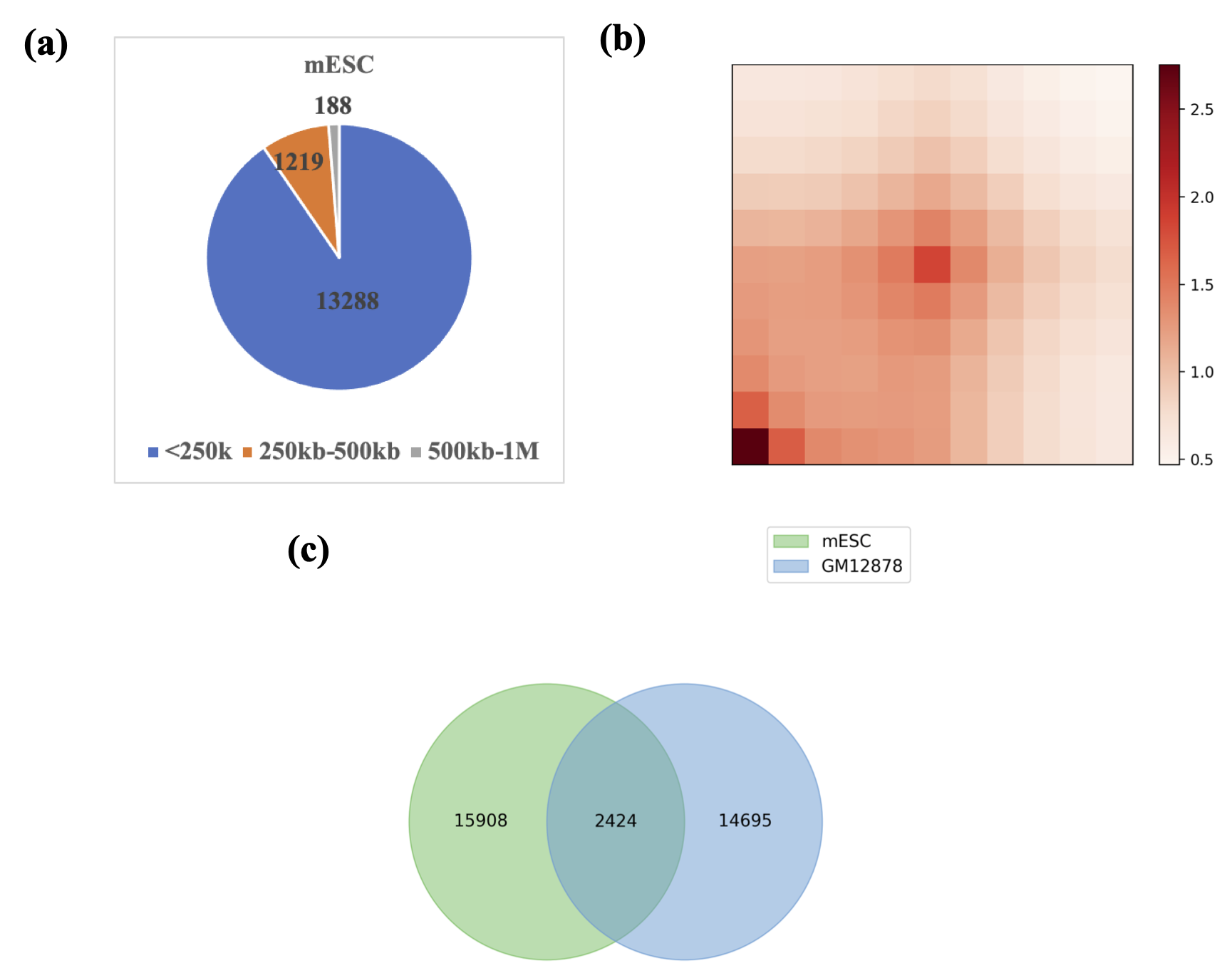

Supplement: S10 Fig — (a) Distance distribution of DLoopCaller identified SMC1 HiChIP chromatin loops from Hi-C contact maps in mESC. (b) The APA plots for SMC1 HiChIP chromatin loops in mESC. (c) Venn diagram of DLoopCaller identified SMC1 HiChIP chromatin loops in GM12878 and mESC. (TIF) [file pcbi.1010572.s013.tif]

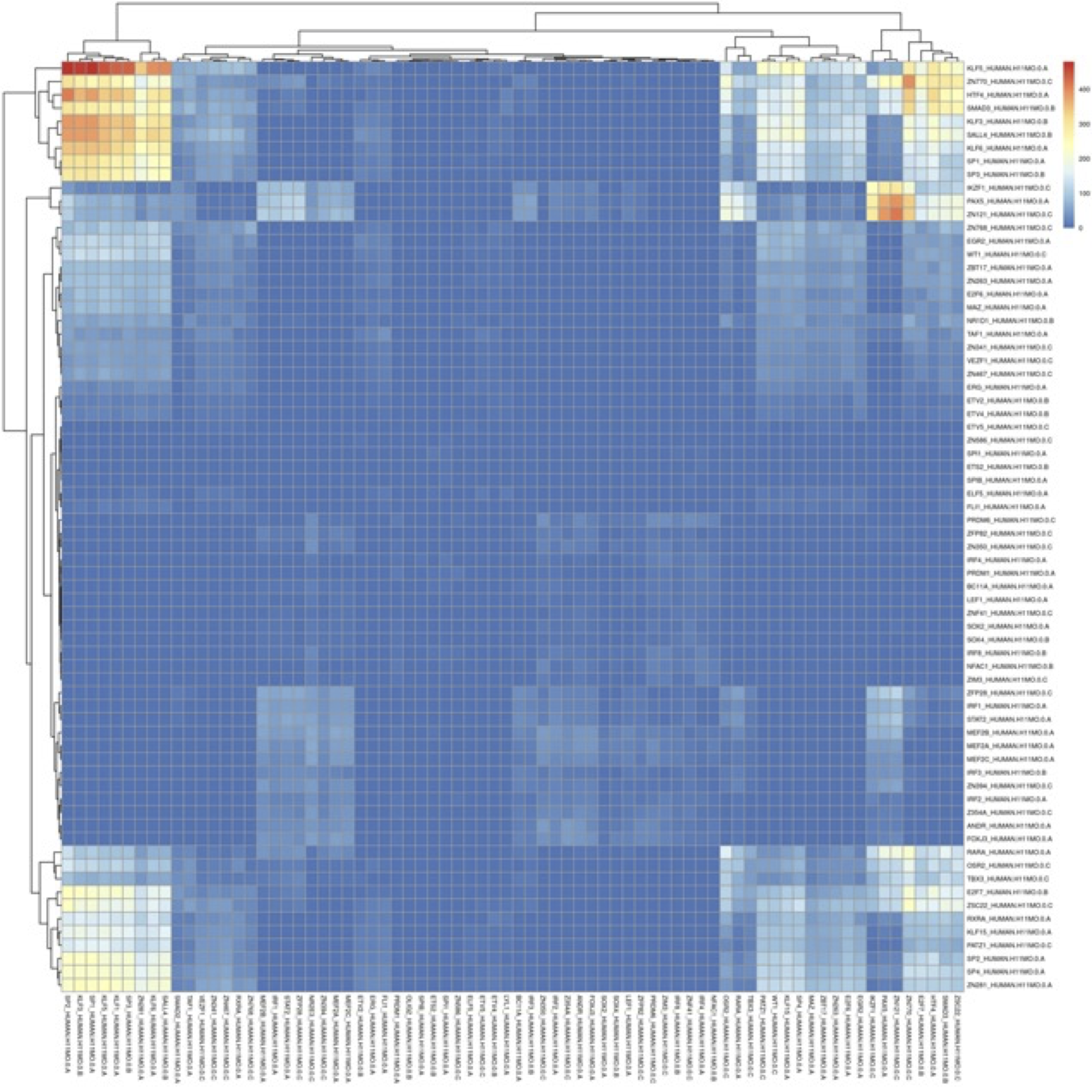

Supplement: S11 Fig — (TIF) [file pcbi.1010572.s014.tif]

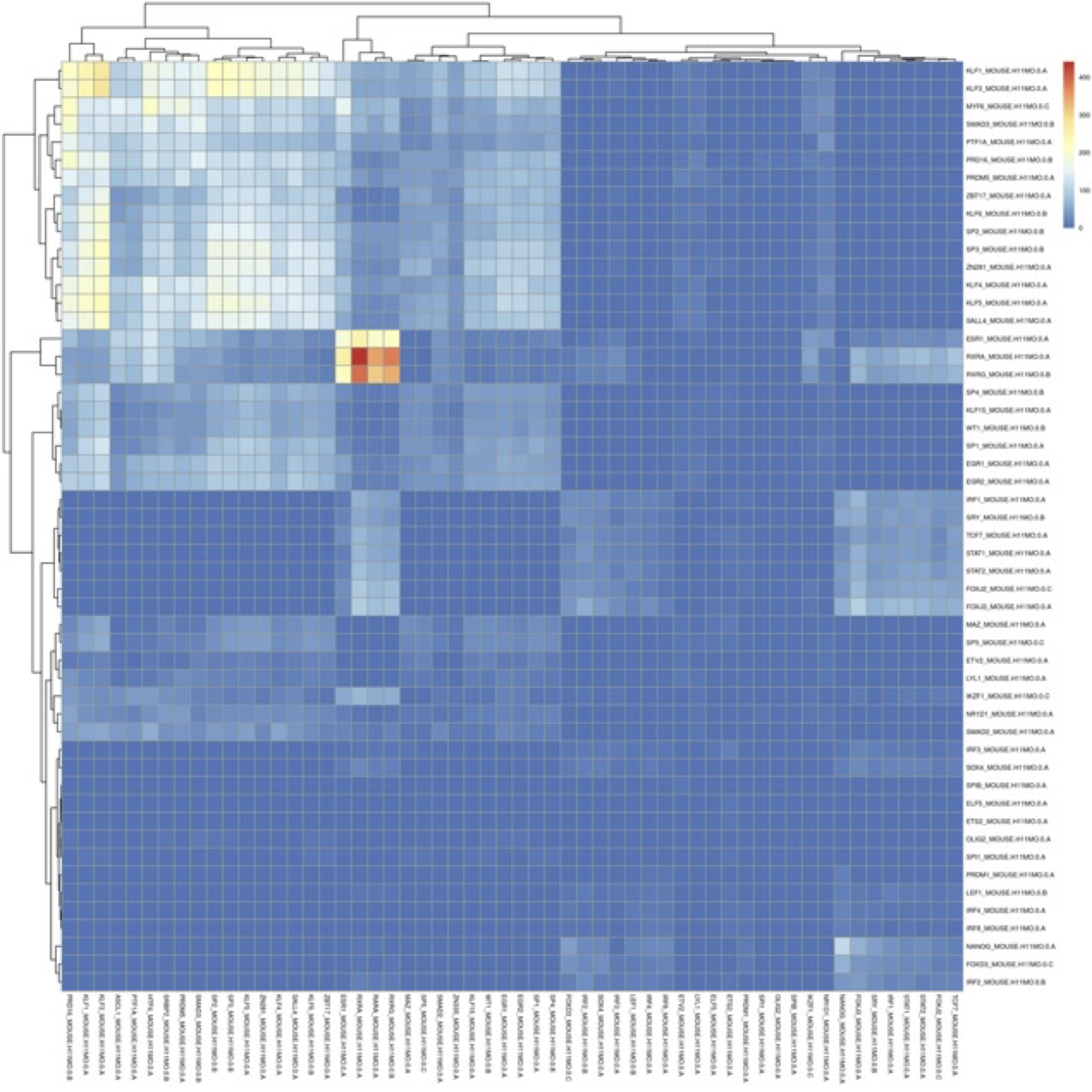

Supplement: S12 Fig — (TIF) [file pcbi.1010572.s015.tif]

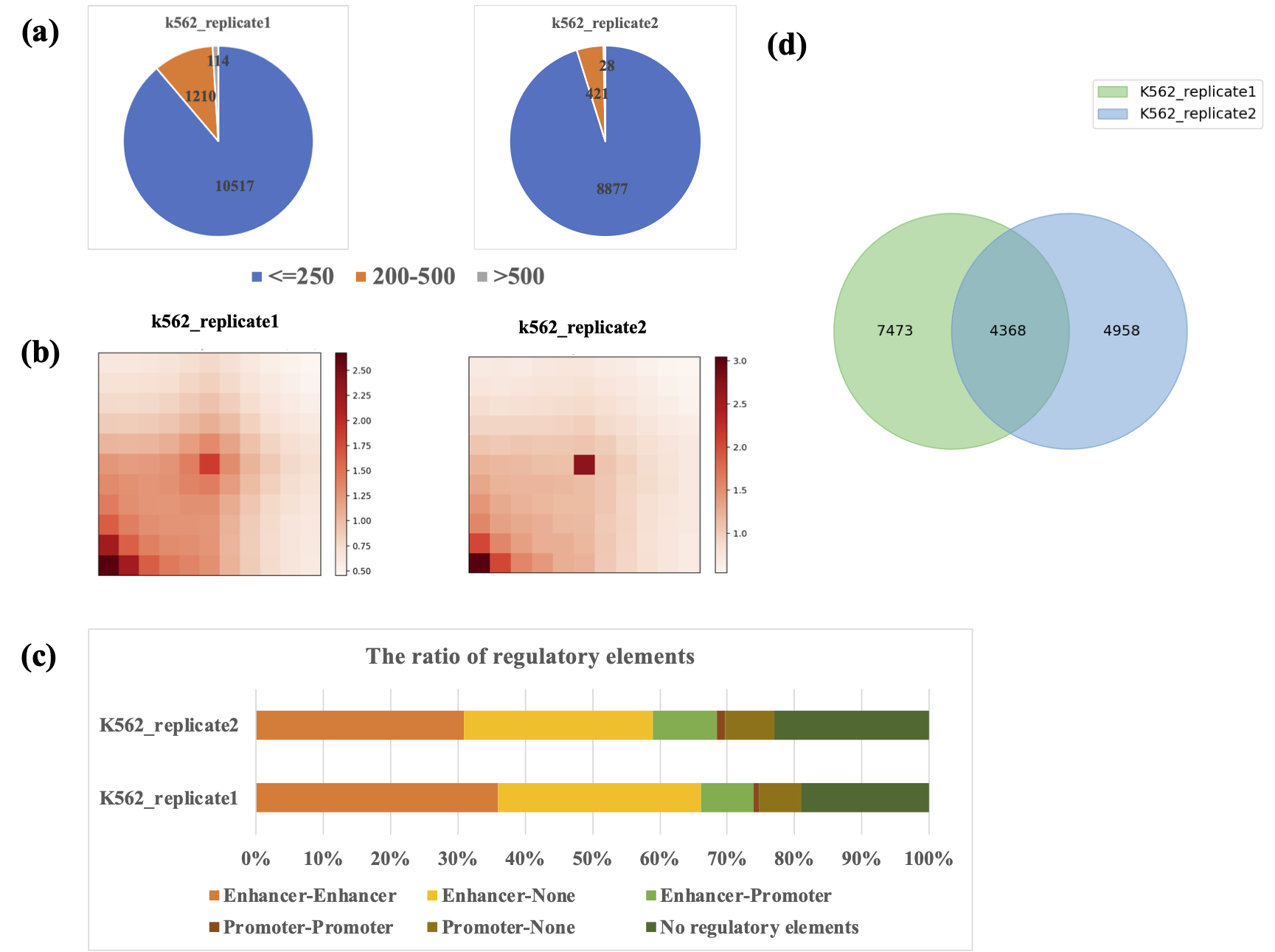

Supplement: S13 Fig — (a) Distance distribution of DLoopCaller identified chromatin loops from Hi-C contact maps by using CTCF ChIA-PET data after training on two replicates of K562; (b) APA plots for DLoopCaller CTCF ChIA-PET loops in two replicates of K562; (c) The proportion of identified chromatin loops types using CTCF ChIA-PET data after training for two replicates of K562; (d) Venn diagram of CTCF ChIA-PET chromatin loops in two replicates of K562. (TIF) [file pcbi.1010572.s016.tif]

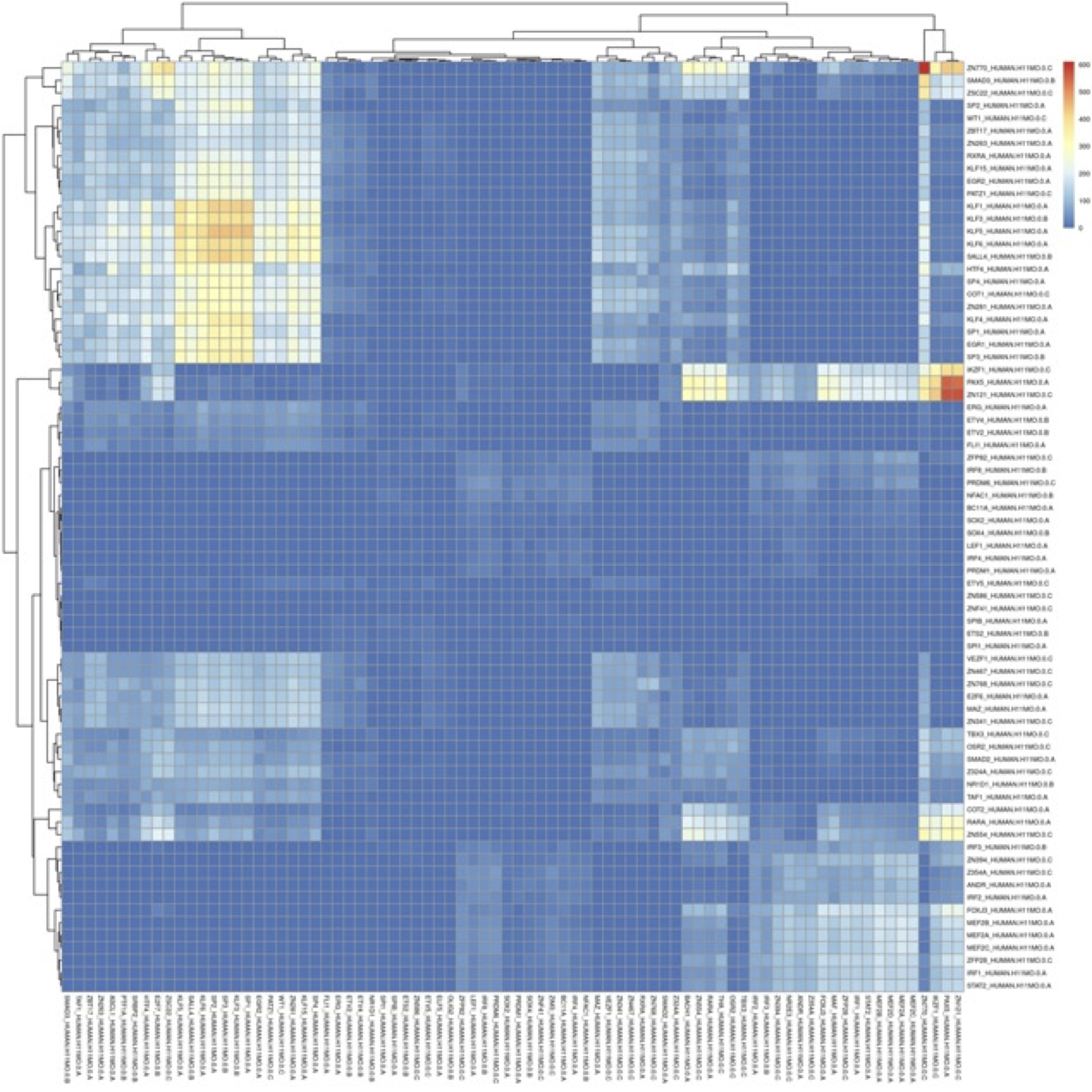

Supplement: S14 Fig — (TIF) [file pcbi.1010572.s017.tif]

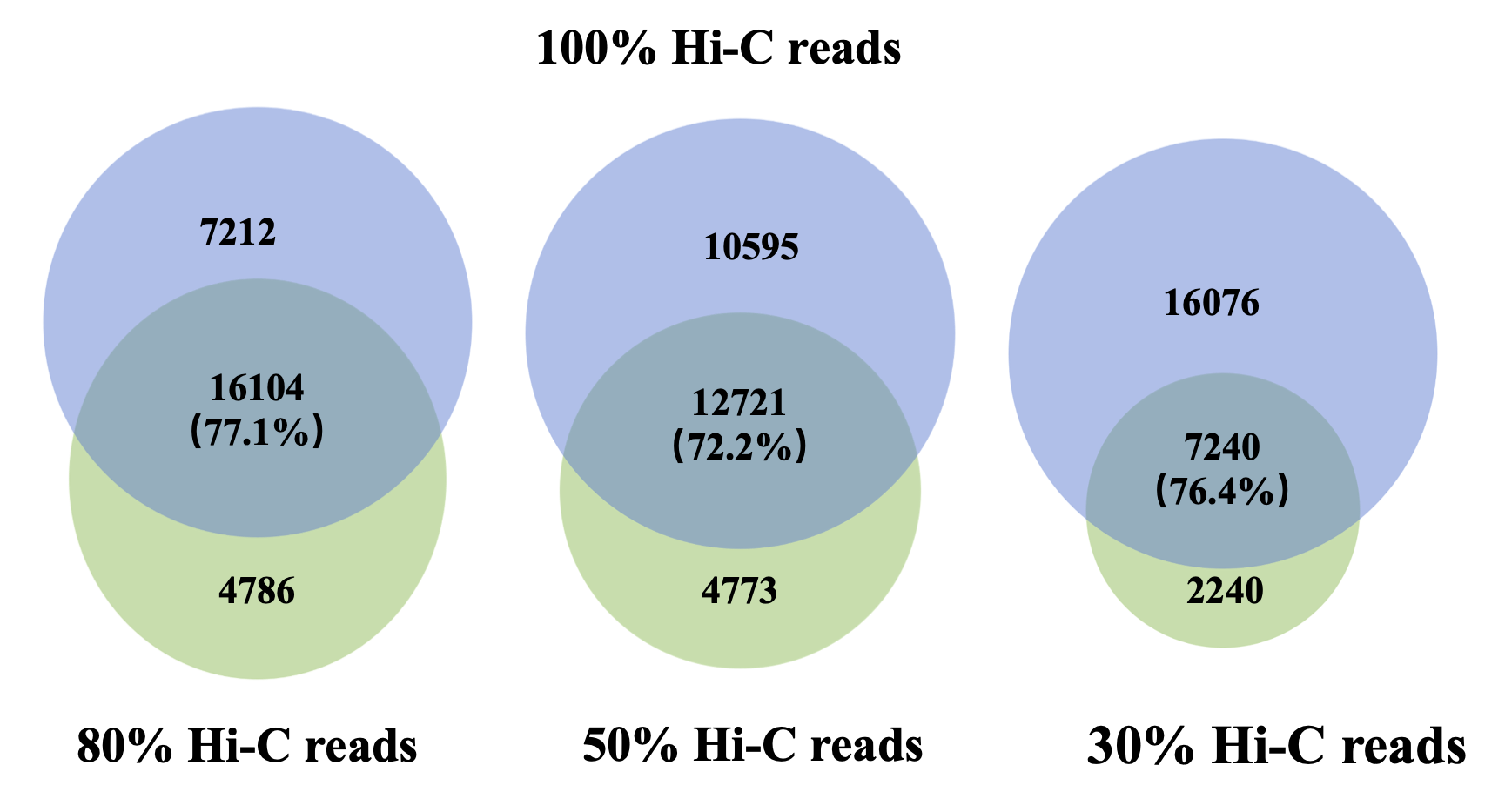

Supplement: S15 Fig — (TIF) [file pcbi.1010572.s018.tif]
